# Human Complement C4B Allotypes and Deficiencies in Selected Cases With Autoimmune Diseases

**DOI:** 10.3389/fimmu.2021.739430

**Published:** 2021-10-26

**Authors:** Danlei Zhou, Michael Rudnicki, Gilbert T. Chua, Simon K. Lawrance, Bi Zhou, Joanne L. Drew, Fatima Barbar-Smiley, Taylor K. Armstrong, Miranda E. Hilt, Daniel J. Birmingham, Werner Passler, Jeffrey J. Auletta, Sasigarn A. Bowden, Robert P. Hoffman, Yee Ling Wu, Wael N. Jarjour, Chi Chiu Mok, Stacy P. Ardoin, Yu Lung Lau, Chack Yung Yu

**Affiliations:** ^1^ Center for Microbial Pathogenesis, Abigail Wexner Research Institute, Nationwide Children’s Hospital, Columbus, OH, United States; ^2^ Division of Rheumatology, Nationwide Children’s Hospital, Columbus, OH, United States; ^3^ Department of Internal Medicine IV – Nephrology and Hypertension, Medical University Innsbruck, Innsbruck, Austria; ^4^ Department of Paediatrics and Adolescent Medicine, Queen Mary Hospital, The University of Hong Kong, Hong Kong, Hong Kong, SAR China; ^5^ Department of Biology & Earth Science, Otterbein University, Westerville, OH, United States; ^6^ Department of Pediatrics, The Ohio State University, Columbus, OH, United States; ^7^ Barbara Davis Center for Childhood Diabetes, University of Colorado, Aurora, CO, United States; ^8^ Department of Internal Medicine, The Ohio State University, Columbus, OH, United States; ^9^ Division of Nephrology and Dialysis, City Hospital, Bolzano, Italy; ^10^ Division of Hematology/Oncology, Nationwide Children’s Hospital, Columbus, OH, United States; ^11^ Division of Endocrinology, Nationwide Children’s Hospital, Columbus, OH, United States; ^12^ Department of Microbiology and Immunology, Loyola University Chicago, Maywood, IL, United States; ^13^ Department of Medicine, Tuen Mun Hospital, Hong Kong, Hong Kong, SAR China

**Keywords:** Anti-NMDA receptor encephalitis, complement C4 polymorphism, C4B mutations, gene copy number variation, race-specific variations, RCCX modules, systemic lupus erythematosus, type 1 diabetes

## Abstract

Human complement C4 is one of the most diverse but heritable effectors for humoral immunity. To help understand the roles of C4 in the defense and pathogenesis of autoimmune and inflammatory diseases, we determined the bases of polymorphisms including the frequent genetic deficiency of C4A and/or C4B isotypes. We demonstrated the diversities of C4A and C4B proteins and their gene copy number variations (CNVs) in healthy subjects and patients with autoimmune disease, such as type 1 diabetes, systemic lupus erythematosus (SLE) and encephalitis. We identified subjects with (a) the fastest migrating C4B allotype, B7, or (b) a deficiency of C4B protein caused by genetic mutation in addition to gene copy-number variation. Those variants and mutants were characterized, sequenced and specific techniques for detection developed. Novel findings were made in four case series. First, the amino acid sequence determinant for C4B7 was likely the R729Q variation at the anaphylatoxin-like region. Second, in healthy White subject MS630, a C-nucleotide deletion at codon-755 led to frameshift mutations in his single *C4B* gene, which was a private mutation. Third, in European family E94 with multiplex lupus-related mortality and low serum C4 levels, the culprit was a recurrent haplotype with *HLA-A30, B18* and *DR7* that segregated with two defective *C4B* genes and identical mutations at the donor splice site of intron-28. Fourth, in East-Asian subject E133P with anti-NMDA receptor encephalitis, the *C4B* gene had a mutation that changed tryptophan-660 to a stop-codon (W660x), which was present in a haplotype with HLA-DRB1*04:06 and B*15:27. The W660x mutation is recurrent among East-Asians with a frequency of 1.5% but not detectable among patients with SLE. A meticulous annotation of *C4* sequences revealed clusters of variations proximal to sites for protein processing, activation and inactivation, and binding of interacting molecules.

## Introduction

**Figure 1:**
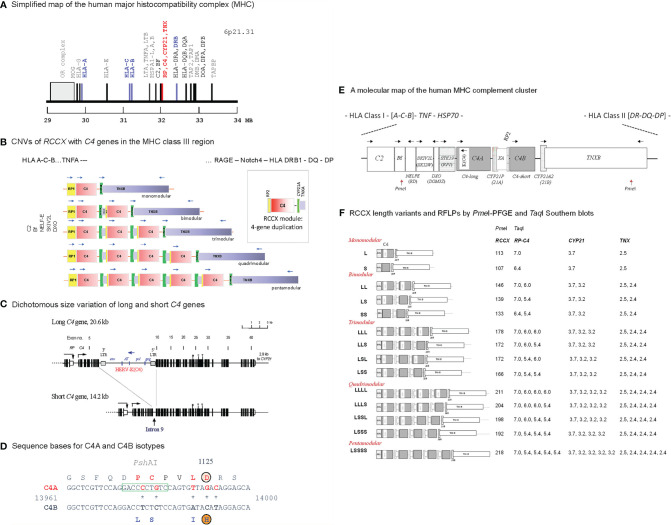
Genetic diversities of complement C4 - concepts and strategies of determination. **(A)** A simplified genetic map of the human leukocyte antigen (HLA) on the short arm of chromosome 6 highlighting genes relevant to immune response and inflammation. **(B)** Gene copy number variations (CNVs) of the *RP-C4-CYP21-TNX* (RCCX) modules (3, 5, 6). Haplotypes with one to five RCCX modules are shown. Blue, horizontal arrows above genes represent gene orientations and directions of transcription. The boxed structure at the right corner represents an RCCX module that is being duplicated. **(C)** Exon-intron structures with dichotomous size variation of long and short *C4* genes. Symbols over exons depict locations encoding for the thioester bond (exon 24), C4A and C4B isotypic residues (exon 26), and major Rodgers and Chido blood group antigens (exon 28) (7–9). An inverted arrow under intron 9 of the short *C4* gene indicates the corresponding integration site for the endogenous retrovirus HERV-K(C4) in the long genes. **(D)** Differentiation of *C4A* and *C4B* genes and protein isotypes. Amino acid residues specific for C4A are shown in red fonts; amino acid residues specific for C4B are shown in blue fonts. *Psh*AI restriction enzyme cleavage site specific for *C4A* is boxed. **(E)** A molecular map of gene organizations of the MHC-complement gene cluster with a bimodular *long-short* (LS) RCCX structure. Horizontal arrows stand for transcriptional orientations. The two *Pme*I cleavage sites that encompass the entire RCCX modules in pulsed field gel electrophoresis (PFGE) are indicated (10). **(F)** Structures of fourteen known RCCX length variants. The *Pme*I restriction fragment sizes resolved by PFGE are listed. Detailed experimental protocols for RCCX genotyping and C4 variants can be found in reference (10). The fragment sizes of *Taq*I restriction fragments for the *RP-C4*, *CYP21* and *TNX* are shown on the right panel. Note that some individuals may contain duplicated copies of functional *CYP21B (*renamed to *CYP21A2)*, or rearranged *TNXA*.

Complement C4 is an effector protein for innate and adaptive humoral immunity (1–3). Activated C4 anchors the formation of C3 and C5 convertases and propagates the classical and lectin pathways (see **Supplementary Figure S2**). During this process, anaphylatoxins are generated to attract inflammatory cells migrating to the site of complement activation, immune complexes are opsonized for phagocytosis by myeloid cells, and membrane attack complexes are formed to lyse cellular or microbial targets (4). Human complement C4 features quantitative and qualitative diversities, which are mainly attributable to the frequent gene copy-number variations (CNVs) with two to ten copies of long or short *C4* genes in a diploid genome (**Figure 1**), leading to a large range of serum C4 protein levels among different individuals (11–14).

CNVs of complement *C4* genes are modular and concurrent with three neighboring genes encoding for the serine/threonine kinase RP (also known as STK19) at the upstream region, and cytochrome P450 steroid 21-hydroxylase (CYP21) and extracellular matrix tenascin *TNX* at the downstream region (**Figure 1**). This phenomenon is known as RCCX modular duplications (15–18). In a duplicated RCCX module, only the *C4* gene is intact. The *RP* and the *TNX* are incomplete gene fragments known as *RP2* and *TNXA*. The duplicated *CYP21* is mostly a pseudogene (*CYP21A* or *CYP21P*) with multiple deleterious mutations. Each duplicated *C4* gene may be a long gene with the endogenous retrovirus HERV-K(C4) integrated into its intron 9 (7), or a short gene without this retroviral element. Each *C4* gene may code for a C4A or a C4B protein.

The C4 proteins are highly polymorphic with multiple variants for two isotypes: the acidic C4A and the basic C4B with fast and slow migration properties, respectively, in an agarose allotyping gel that resolves protein variants based on gross difference in electric charge (19–21). C4A and C4B proteins have differential binding affinities and hemolytic activities towards their molecular targets, which form covalent amide bond with activated C4A or covalent ester bond with activated C4B (22, 23). While occurring infrequently, patients with a complete genetic deficiency for both C4A and C4B are usually inflicted with inflammatory and/or infectious diseases, and with chronic autoimmune disease such as systemic lupus erythematosus (SLE) (3, 24). Low gene copy-number, low serum protein levels, or a deficiency of the C4A isotype are prevalent among patients with SLE in various racial groups (11, 12). Intriguingly, among patients with SLE or antiphospholipid antibodies, those with relatively higher C4 protein levels or with high copy-number of *C4B* genes are associated with increased risk for chronic hypertension and thromboses (25, 26). Higher gene copy-number of *C4A* or a genetic deficiency of *C4B*, is a risk factor for neurologic or psychiatric disorder such as schizophrenia (27–29).

Complete deficiency for both C4A and C4B are usually caused by private and nonsense mutations that totally abrogate C4 protein translation (30, 31). In several cases of complete C4A and C4B deficiencies, both *C4A* and *C4B* genes were present but mostly acquired identical nonsense mutations in both genes (24, 32–34). On the deficiency of C4A, the absence of a *C4A* gene in the HLA haplotype with *A1 B8* and *DR3* (DRB1*03:01) (18, 35–37), or a 2-bp insertion at codon 1232 (formerly 1213 when the N-terminus residue of the mature protein was assigned as number 1) leading to nonsense mutations are recurrent causes, mostly in HLA haplotypes with *B60* and *DR6*, and *B8* and *DR3* or *DR6* (38). Nonsense mutations specific for *C4B* genes had not been identified. Elucidations of the molecular basis for the phenotypic diversities of complement C4A and C4B may help determine their physiologic roles in health and disease.

Here we present results on our pursuits to elucidate the molecular bases of complement C4 polymorphisms and deficiencies. Among them are a subject with fast-migrating C4B that is detectable in many individuals of European ancestry, and three different cases of C4B deficiencies in healthy subjects and patients with SLE or anti-NMDAR encephalitis. Novel findings of the polymorphic variant for C4B7 and mutations causing C4B deficiency were then extended to large study populations to evaluate their prevalences.

## Study Populations, Materials, and Methods

### Human Subjects and Blood Samples

Human subjects were recruited with informed consents according to protocols approved by Institutional Review Board of the Nationwide Children’s Hospital (NCH), Columbus, Ohio. Black and White healthy subjects for this study were recruited in Columbus, Ohio of the US. They self-reported to have no genetic or autoimmune disease. Genomic DNA and EDTA-plasma samples were prepared from peripheral blood samples, as described previously (5, 10, 17, 18).

A healthy child with an unusual C4B allotype C4B7 (HC74) and a young adult with complement C4B deficiency (MS630) were recruited in Columbus, Ohio through our ongoing studies on complement polymorphisms. These two individuals were White but de-identified during the recruitment process and therefore their family members were not studied.

Type 1 diabetes (TD) patients described in this manuscript were White and recruited at the Endocrinology and Diabetes Clinics of the NCH. TD patients were studied to illustrate the extent of C4 protein polymorphisms. A total of 340 White TD patients from the NCH were recruited, results for six of those patients were selected for presentation because they exhibited distinct C4 polymorphic variants.

Clinical data of an SLE patient (E94P) and her family members including clinical laboratory C4 protein levels, antinuclear antibody (ANA), anti-double stranded DNA levels, and HLA class I and Class II gene alleles were determined at Medical University of Innsbruck, Austria. With informed consents, extended family members donated blood samples for this study.

Blood samples and clinical data from a patient with anti-N-methyl-D-aspartate (anti-NMDA) receptor encephalitis E133P and his family members were recruited at the Queen Mary Hospital, The University of Hong Kong, SAR, China. A detailed clinical case report of this patient has been published recently (39).

Control samples for European and East-Asians were (a) recruited from the staff members of the Nationwide Children’s Hospital or at annual Asian Festivals during Memorial holiday weekends in Columbus OH, with informed consents, or (b) processed from expired blood samples from volunteers who donated blood to the Red-Cross in Hong Kong. Genomic DNA and plasma samples from East Asian SLE patients for screening of W660x mutation originated from Hong Kong were from Queen Mary Hospital (11) and Tuen Mun Hospital, Hong Kong SAR. Clinical features for East-Asian SLE samples studied for complement *C4* gene CNVs can be found in reference (11).

### *RP-C4-CYP21-TNX* Modules and *C4* Gene Copy Numbers

RCCX modular variation was determined by digesting genomic DNA samples (5 µg per sample) with the restriction enzyme *Taq*I, resolving the restriction fragments by agarose gel electrophoresis, transferring the fragments to nylon membranes according to Southern’s procedure and hybridizing the membranes with DNA probes corresponding to the intergenic region between *RP* and *C4*, to steroid 21-hydrodxylase *CYP21*, and to the 3’ region of tenascin *TNX* (**Figure 1**) (10, 15, 40, 41).

The *RCCX* haplotypes were further confirmed by sub-megabase scale physical mapping. Genomic DNA from leukocytes was embedded in agarose gel plugs, digested with the restriction enzyme *Pme*I, resolved by pulsed field gel electrophoresis (BioRad Chef Mapper), transferred to nylon membranes according to Southern’s procedure, and hybridized with a C4d-specific probe for X-ray film autoradiography (5, 10).

The relative copy numbers of *C4A* and *C4B* genes were determined by hybridization of Southern blots for genomic DNA digested by *Psh*AI and *Pvu*II, with a *C4d*-specific probe for all subjects reported here. As genomic DNA for patient E94P was limited in quantity, her *C4A* and *C4B* gene copy numbers were determined by quantitative real-time PCR, as described previously (41). The real-time PCR method yielded identical results to those derived from Southern blots.

### C4A and C4B Protein Allotypes by Immunofixation

C4A and C4B protein allotypes were determined by immunofixation. Briefly, EDTA-plasma samples previously stored at -80°C were retrieved and were digested with neuraminidase from *Clostridium perfringes* (Millipore Sigma, St Louis MO) to eliminate glycosylation heterogeneity, then with carboxyl peptidase B (Calzyme Laboratories, Inc, San Luis, Obispo, CA) to eliminate irregularities of the C4 α and β chain carboxyl termini created by proteolytic cleavages between the junctions of β−α and the α−γ chains, followed by high voltage agarose gel electrophoresis to resolve protein molecules based on gross differences in electric charge. C4A and C4B protein in agarose gel were fixed with goat anti-human C4 antiserum (Complement Technology Inc. Tyler TX), blotted to remove diffusible proteins and stained with SimplyBlue Safe stain (Invitrogen) (10, 20).

### Long-Range PCR for *C4B* Genes and DNA Sequence Analyses

#### Long *C4B* Genes in HC74, MS630 and E133P

The long *C4B* mutant genes for each of these three subjects were amplified in four fragments using long range PCR. The first fragment was 2.3 kb in size and spanned from the promoter region (C4 Pro1) of the *C4* gene to exon 1 to exon 9. It was generated by the forward primer C4-Pro1: 5’-CAA GGT CCA GAG TCA ACT CTG C-3’ and a reverse primer at the 3’ end of exon 9 or C4-E9.32: 5’-CCT GGA GAC TAA TGA TGG CTG C-3’. The PCR conditions were one cycle at 94°C for 3 min; 35 cycles at 94°C for 45 s, 64°C for 60 s, and 72°C for 5 min; and one cycle at 72°C for 10 min. This PCR fragment did *not* differentiate between *C4A* and *C4B* genes.

The second fragment was 4.9 kb in size and covered exons 10–26 specific for *C4B*. It was amplified by a forward primer from exon 10 or C4-E10.5: 5’-GGA GGC AGA GCT CAC ATC CTG-3’ and a reverse primer from exon 26 with C4B specific sequence or C4B-UP: 5’-GCA CCT GCA TGC TCC TAT GTA TC-3’. The PCR conditions were one cycle at 94°C for 3 min; 35 cycles at 94°C for 45 s, 64°C for 60 s, and 72°C for 9 min; and one cycle at 72°C for 10 min.

The third fragment was 3.5 kb in size, corresponding to exons 26–35 specific for C4B, and was produced using a forward primer specific for C4B (at the 3’ end of exon 26) or C4B-down: 5’-GAC CTC TCT CCA GTG ATA CAT AG-3’, and a reverse primer at the 3’ end of exon 35 or C4-35.3: 5’-GAG TCA AAA TAC AGC AGG ACG TG-3’ The PCR conditions were one cycle at 94°C for 3 min; 35 cycles at 94°C for 45 s, 64°C for 60 s, and 72°C for 9 min, one cycle at 72°C for 10 min.

The fourth fragment was 3.6 kb in size, corresponding to exons 33–41 that is common to both *C4A* and *C4B*, and was produced using a forward primer at the 3’ end of exon 33 or C4- 33.5: 5’-GGA AGC AAA CGA GGA CTA TGA GG-3’, and a reverse primer at the 3’ end of exon 41 or C4-41.32: 5’-CAG CTT CAT GGT TCC CAG GTT C-3’. The PCR conditions were one cycle at 94°C for 3 min; 35 cycles at 94°C for 45 s, 64°C for 60 s, 72°C for 9 min; and one cycle at 72°C for 10 min (33).

All PCR were performed using the Failsafe PCR amplification kit (Epicentre Technologies, Madison, WI). The four DNA fragments were resolved using low gelling temperature agarose gel electrophoresis, and DNA fragments were excised from gel and purified using a PCR purification kit (Qiagen, Valencia, CA). They were sent to the Eurofins Genomics (Chicago, IL) for Sanger’s DNA sequencing with appropriate primers.

#### The Short *C4B* Genes in E94

This multiplex family with SLE-related mortality had members consisting of a special HLA haplotype *A18 B18 DR7* that is identical to patients with complete complement C4 deficiency, which was caused by a donor site mutation at intron-28 of all *C4* genes (33). Thus, we investigated whether members of the E94 family had the same intron-28 splice site mutation of the *C4* genes. An approximately 300 bp PCR product was produced using primers I27F (5’-CAA GAC CCT CCT CCC GTT TTC-3’) in combination with the reverse primer MBO-28R (5’-ACT TCA TTC CTC CTC TGA GTC -3’). The PCR conditions were one cycle 3 min at 94°C; 35 cycles of 30 s at 94°C, 30 s at 62°C and 45 s at 72°C, followed by a final extension of 5 minutes at 72°C. PCR products were purified and subject to Sanger’s DNA sequencing as described earlier (33).

### Verifications of a Polymorphism for Fast-Migrating C4B7 and Novel C4B Nonsense Mutations

DNA sequences were analyzed through standard Nucleotide BLAST with the NCBI website to identify mutations or polymorphic sites. Reference DNA sequence for human *C4B* and mutant sequences were compared to identify specific restriction sites using EMBOSS program “remap”. Afterwards, specific genomic DNA fragments containing the putative mutations and restriction sites were amplified by PCR, followed by restriction enzyme digestion.

#### HC74

To detect the G→A transition causing R729Q variation in exon 17 of *C4B* gene in HC74 sample, primers C4 E16.5F 5’-GTT GCT GGT CTC AAG GGG TC-3’ and C4 E18.3R 5’-GCC GGG CCG GCA CAC ACT CTC-3’ were used for DNA amplification. PCR conditions were identical to the C-deletion determination (see below). PCR products were digested overnight with restriction *Nci*I enzyme at 37°C and resolved by electrophoresis with a 1.2% agarose gel (33).

#### MS630

To detect the C-nucleotide deletion in exon 17 of the *C4B* gene (L755 ΔC) in MS630 sample, primers C4 16.5F and C4 18.5R CCTGCAGGATCTCCAGGGCTG were used for DNA amplification. PCR was performed using the Failsafe PCR amplification kit and conditions were one cycle at 94°C for 3 min; 35 cycles at 94°C for 30 s, 62°C for 45 s, and 72°C for 1 min; and one cycle at 72°C for 10 min. PCR products were digested with restriction enzyme *Eco*NI enzyme at 37°C for 3 hr and resolved by electrophoresis with a 1.5% agarose gel. Genomic DNA for more than 500 White and Black subjects was tested for this mutation.

#### E133

Sequence-specific Primer PCR (SSP-PCR) was used to determine the G→ A mutation in exons 15 for W660x of the *C4B* gene using primers C4-14.5F 5’-CTG GAG CTC AGC GTG GAC GGT-3’ and C4-18.3R in E133 family. PCR was performed using the Failsafe PCR amplification kit. The PCR conditions were one cycle at 94°C for 3 min; 35 cycles at 94°C for 30 s, 62°C for 45 s, and 72°C for 75 s; and one cycle at 72°C for 10 min. The PCR products were digested overnight with restriction enzyme *Acc*I at 37°C and resolved by electrophoresis with a 1.2% agarose gel.

### *HLA - A*, *B*, and *DRB1* Genotyping

Custom genotyping of *HLA* class I genes *A* and *B* and class II gene *DRB1* were performed by the Barbara Davis Center for Childhood Diabetes at University of Colorado Anschutz Medical Campus, Aurora, CO.

## Results

### Polymorphisms of C4B and C4A Proteins

**Figure 2:**
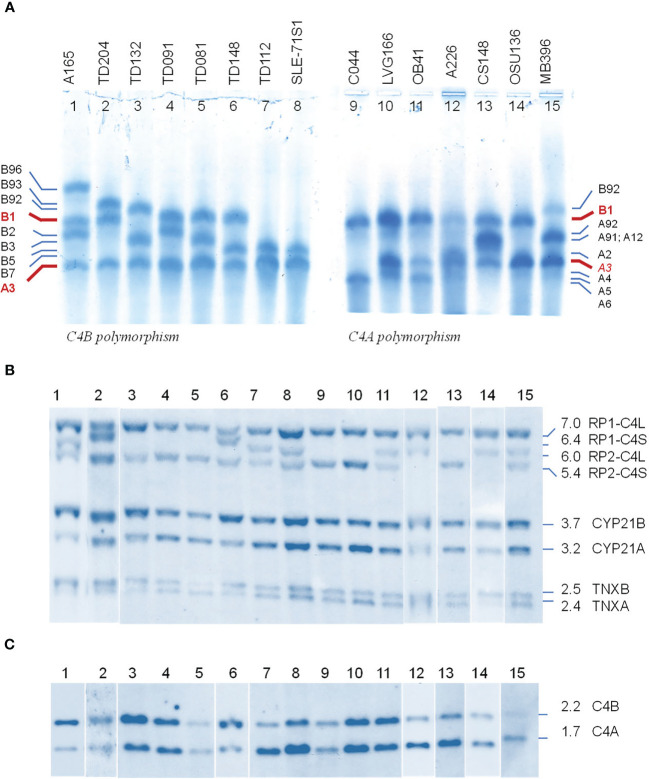
Polymorphic variants, RCCX modules and gene copy number variations of human complement C4B and C4A - from phenotypes to genotypes. **(A)** Polymorphisms of C4 protein. EDTA-plasma from 15 human subjects were treated with neuraminidase and carboxypeptidase B, subjected to high voltage agarose gel electrophoresis to resolve C4 plasma proteins based on gross differences in electric charge, immunofixed with goat antiserum against human C4, blotted to remove diffusible proteins and then stained. The most common form of C4 allotypes are B1 and A3 (red fonts). The left of *panel* **(A)** showed common variants of C4B. The right of *panel* **(A)** showed common variants of C4A. Band intensities in each *lane* reflected the relative expression levels of various protein allotypes. **(B)** *Taq*I restriction fragment length polymorphism (RFLP) of DNA samples from the same subjects to demonstrate RCCX modular variations. **(C)** *Psh*AI-*Pvu*II RFLP to elucidate the relative dosage of *C4B* and *C4A* genes. Please refer to of **Figure 1F** for interpretation of *long* and *short C4* genes in linkage with *RP1* or *RP2*. Definitive assignments of C4A and C4B protein allotypes in each subject often require parallel genotyping experiments to determine *RCCX* modular variations and *C4A* and *C4B* gene copy numbers as shown here.

We selected fifteen EDTA-plasma samples from healthy subjects and patients with immune-mediated disorders to demonstrate the phenotypic diversities of C4B and C4A allotypes based on gross differences in electric charge for the native C4 proteins (19–21). As shown in **Figure 2A**, the samples were posited to demonstrate the differential mobilities of polymorphic variants: basic and slower C4B allotypes in the left panel (*lanes* 1 to 8), and acidic and faster C4A allotypes on the right panel (*lanes* 9 to 15). The most common variants for C4B and C4A are B1 and A3, respectively. *Lanes* 1 to 3 showed samples that also consisted of C4B variants migrating slower than B1, which were B96, B93 and B92. *Lanes* 1 and 3 to 8 presented samples with C4B variants migrating faster than B1, which are B2, B3, B5 and B7. It is of interest to note that B96 in *lane* 1 (A-165) represents the slowest migrating C4B allotype, while B7 in *lanes* 6 (TD148) and 8 (SLE-71S1) represents the fastest migrating C4B allotype. Samples from patients with type 1 diabetes (TD) had multiple C4B variants that migrated between A3 and B1 (*lanes* 2-7, **Figure 2A**).

Of the C4A variants, *lanes* 9-11 with samples for C044, LVG-166 and OB41 had faster migrating variants than A3, which were A6, A4 and A5, respectively. *Lane* 12 showed a subject (A-226) expressing A2 that migrated just slower than A3. *Lane* 13 for CS148 had allotypes A91 and A12, which migrated to the middle between A3 and B1. These two allotypes had differential expression levels, with A91>>A12. *Lane* 15 showed another subject expressing A91 in addition to A3.

The call for the protein allotypes migrating between C4B1 and C4A3 would not be accurate without data from parallel genotyping experiments. The *C4* gene copy number in each subject were elucidated by *Taq*I RFLP for length variants of RCCX modules with long or short *C4* genes (7) in the first locus and the subsequent loci in the MHC class III region (**Figure 2B**; please also refer to **Figure 1E** for RFLP data interpretation). The C4A and C4B isotypic assignments were corroborated by results of *Psh*AI/*Pvu*II RFLP that differentiated *C4A* and *C4B* genes (**Figure 2C**). For example, on the protein allotype of CS148 (*lane* 13), it was clear for the presence of B1 and A3. However, there were no clear ways to assign the C4A or C4B allotypes that migrated to the middle of this *lane*. Our *Taq*I-RFLP revealed the presence of LS/LS modules for RCCX with four copies of *C4* genes (**Figure 2B**). Meanwhile, *Psh*AI/*Pvu*II RFLP revealed the presence of both *C4A* and *C4B* genes but the dosage of *C4A* was much greater than *C4B* and therefore the logical assignment would be three *C4A* and one *C4B*. One of the *C4A* genes coded for A3 and the other two were responsible for the protein allotypes that migrated to the middle, which were therefore assigned A91 and A12. An interpretation of the RCCX haplotypes, copy numbers of total *C4*, *C4A* and *C4B* genes and their protein allotypes for the fifteen samples presented in **Figure 2** is shown in **Table 1**.

**Table 1:** Genotypes and phenotypes of complement C4B and C4A for subjects shown in **Figure 2**.

No.	Code	RCCX	*C4T*	*C4B*	*C4A*	C4B protein	C4A protein
**1**	A165	LL/LS	4	3	1	B96B1B2	A3
**2**	TD204	LS/S	3	2	1	B93B1	A3
**3**	TD132	LS/L	3	2	1	B92B3	A3
**4**	TD091	LS/LS	4	2	2	B1B2	A3A3
**5**	TD081	LS/LS	4	2	2	B1B3	A3A3
**6**	TD148	LS/S	3	2	1	B1B7	A3
**7**	TD112	LL/LS	4	1	3	B5	A3A3A3
**8**	SLE- 71S1	LL/LS	4	1	3	B7	A3A3A3
**9**	C044	LS/LS	4	2	2	B1B1	A6A6
**10**	LVG166	LSS/ LS	5	2	3	B1B1	A3A3A4
**11**	OB041	LL/LS	4	2	2	B1B1	A3A5
**12**	A226	LL/L	3	1	2	B1	A2A3
**13**	CS148	LS/LS	4	1	3	B1	A91A12A3
**14**	OSU136	LL/L	3	1	2	B1	A3A3
**15**	MB396	LL/LS	4	1	3	B92	A3A3A91

RCCX, RP-C4-CYP21-TNX module.

### Molecular Basis for the Fast Migrating C4B7 (HC74)

**Figure 3:**
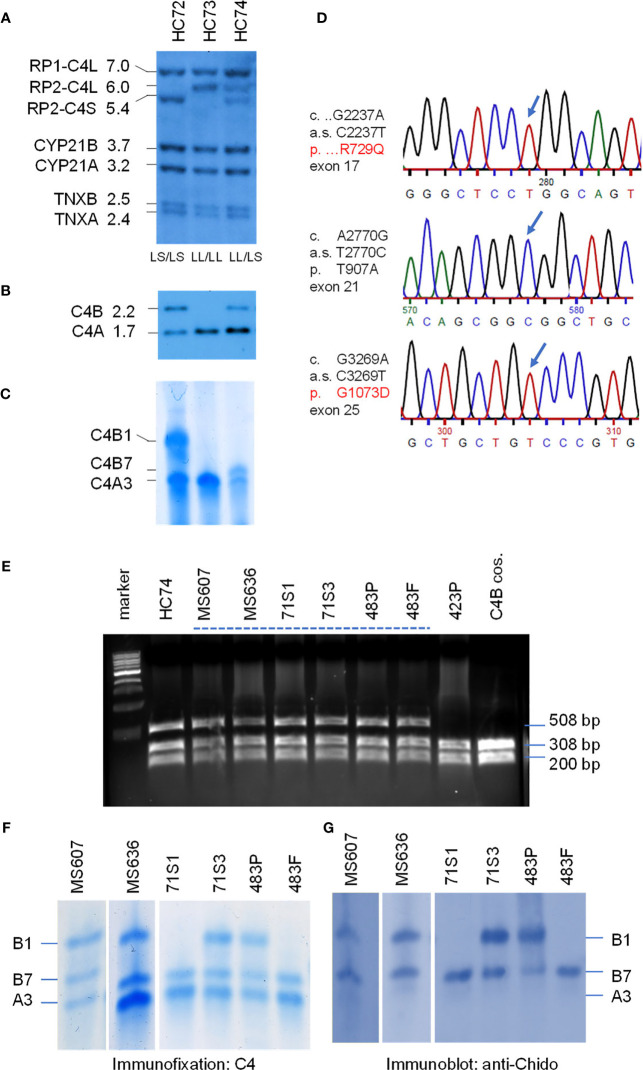
Genotype and phenotype analyses of the fast migrating C4B7 (HC74). **(A)** Southern blot analysis for *RP-C4-CYP21-TNX* (RCCX) modular structures of three White healthy subjects. Genomic DNA samples were digested with *Taq*I restriction enzyme, blotted to nylon membrane and hybridized to ^32^P-labeled specific genomic probes spanning the RCCX to characterize the quantitative variation of *C4* long or short genes linked to *RP1* or *RP2*, the relative dosage of *CYP21B* and *CYP21A*, and the relative dosage of *TNXA* and *TNXB*. **(B)** *Psh*AI/*Pvu*II Southern blot with radioactive C4d probe to determine the relative dosage of *C4B* and *C4A* in the same subjects. **(C)** Immunofixation of C4A and C4B protein from EDTA-plasma resolved by high voltage agarose gel electrophoresis and reacted with polyclonal antiserum against human C4. **(D)** DNA sequences showing three variants identified for HC74 that contributed to R729Q (*upper panel*), T907A (*middle panel*) and G1073D (*lower panel*). **(E)** *Nci*I RFLP to detect DNA sequence change for R729Q in *C4* genes of eight human subjects and a negative control. DNA fragments of 508-bp from both *C4A* and *C4B* genes were amplified, digested with *Nci*I and resolved by agarose gel electrophoresis. The DNA samples for the Q729 variant were resistant to the *Nci*I digest. **(F, G)** Immunofixation **(F)** and immunoblot **(G)** of C4 protein allotypes from six samples with C4B7 identified by PCR in *panel* **(E)**. Fast migrating B7 allotypes were observed in all six samples and they reacted with anti-Chido monoclonal antibodies which are mostly associated with C4B. Notice that the C4A3 protein shown in *panel* **(F)** did not react with the anti-Chido antibody in *panel* **(G)**. The GenBank accession number for the DNA fragment containing sequence with R729Q in HC74 is NZ203454.

On genotyping and phenotyping experiments for complement C4 among healthy subjects, genomic Southern blot analysis of *Taq*I RFLP revealed that healthy child HC74 had bimodular RCCX haplotypes LL and LS with three long and one short *C4* genes (**Figure 3A**). *Psh*AI-*Pvu*II RFLP suggested that those *C4* genes consisted of three *C4A* and one *C4B* (**Figure 3B**). Protein phenotyping revealed the presence of the prevalent C4A3, and a fast migrating C4B allotype C4B7, which traveled to a position close to that of C4A2 in an agarose protein typing gel (**Figure 3C**).

We chose HC74 to determine the molecular basis of C4B7 because this subject consisted of a single *C4B* gene, which simplified experimental design and data interpretation. The coding regions of the *C4B* gene in HC74 were amplified as four fragments: (*a*) C4-Pro to C4E9-32, 2.3 kb; (*b*) C410.5 to C4B-up, 4.9 kb; (*c*) C4B-down to C4-E35.3, 3.5 kb; and (*d*) C4E33.5 to C4E41.32, 3.6 kb (**Figure 1C**). It was noteworthy that fragments *b* and *c* spanning from exon 10 to exon 26, and exon 26 to exon 35, respectively, were *C4B* specific because *C4B*-specific primers were used for amplifications of those two fragments, while fragments *a* and *d* were common to both *C4A* and *C4B* genes. The amplified DNA fragments were gel-purified and sent to a vendor for Sanger’s sequencing. The DNA sequences were compared with referenced sequences through NCBI-Blast program to identify variant sequences for *C4B* (7, 8, 11, 42).

Three non-synonymous polymorphic sites were identified. Two of them were identified previously, which were A2770G of cDNA sequence and Thr 907 Ala (T907A) for protein sequence, and G3269A of cDNA sequence and G1073D for protein sequence. The third variation was a C2237T change at codon 729. This variant changed Arg-729 (codon CGG) to Gln-729 (codon CAG). This novel R729Q polymorphism converts a positively charged arginine residue to an uncharged but polar glutamine, and abolishes an *Nci*I DNA restriction enzyme cleavage site (CCGGG to CCAGG). Thus, 508-bp DNA fragments spanning from exon 16 to exon 17 were amplified by PCR from eight selected individuals plus a cloned *C4B* gene. The PCR fragments were digested with *Nci*I and resolved by 1.2% agarose gel electrophoresis. Amplified fragments of *C4* genes from samples with R729 would be cleaved to 308-bp and 200-bp, and those with the Q729 (found in HC74) remained intact.

As shown in **Figure 3E**, seven out of the nine samples with fast-migrating protein (selected from a repository of >1500 subjects) contained the Q729-related DNA fragments resistant of *Nci*I cleavage. Among them, six plasma samples were further analyzed with immunofixation (**Figure 3F**) and subjected to immunoblot using anti-Chido monoclonal (C4B-related) antibodies (**Figure 3G**). Again, it was conspicuous that the C4B7 protein migrated substantially faster than C4B1 but was slightly slower than C4A3. As expected, the anti-Chido antibodies reacted with the C4B7 allotypes in addition to the C4B1 allotypes. Thus, the C4B7 allotype was detectable in multiple healthy subjects and a patient with SLE (483P). This R729Q polymorphism is located in the C4a anaphylatoxin-like peptide that is released during C4 activation by C1s of the C1 complex or by MASP2 of the MBL complex. Whether this variation has an effect on C4B activation or the potential activity of C4a are yet to be investigated.

Genotyping of HLA were performed from five selected subjects with complement C4B7 for the highly polymorphic class II gene *HLA-DRB1* that is centromeric to RCCX, and class I genes *HLA-B* and *HLA-A* which are telomeric to the *RCCX* (**Table 2** and **Figure 1**). The genes for C4A3 and B7 segregated with the long-short (LS) haplotype of *RCCX*. Two shared HLA haplotypes appeared among those five subjects. The first group was the linkage of LS: C4-*A3 *B7 with HLA-DRB1*14:54 in HC74, SLE-71S1 and SLE-71S3. The two siblings of this SLE-71 family also shared HLA-B*18:01 and HLA-A*30:02. The second group was the linkage of LS: C4*A3 *B7 with HLA-DRB1*16:01, HLA-B*55:01 and HLA-A*02:01 in the father and patient of the SLE-family 483 (**Table 2**).

**Table 2:** HLA, RCCX and C4 plasma protein concentrations of selected study subjects.

a. Demographics, genotypes and phenotypes												
No.	Sample ID	Ethnicity	SEX	[C4]	DRB1_1	DRB1_2	RCCX-C4_1	RCCX-C4_2	HLA B_1	HLA B_2	HLA A_1	HLA A_2
**1**	HC74	Eur	M	18.4	04:07	14:54	LL: A3A3	LS: A3B7	07:02	08:01	01:01	03:01
**2**	SLE 71S1	Eur	F	43.4	11:03	14:54	LL: A3A3	LS: A3B7	18:01	51:01	03:01	30:02
**3**	SLE 71S3	Eur	M	46.7	14:54	15:01	LS: A3B1	LS: A3B7	18:01	38:01	25:01	30:02
**4**	SLE 483P	Eur	M	10.8	07:01	16:01	LS: A3B7	LS: A3B1	51:01	55:01	02:01	24:02
**5**	SLE 483F	Eur	M	50.3	04:01	16:01	LS: A3B7	LL: A3A3	44:02	55:01	02:01	03:01
**6**	MS630	Eur	M	19.8	04:07	15:01	LL: A3A2	LL: A3Bx	07:02	07:02	03:01	31:01
**7**	E133P	EA	M	7.7	04:05	04:06	LL: A3Bx	L: A3	15:27	46:01	11:01	11:01
**8**	E133M	EA	M	20.2	04:06	12:02	LLS: A3A3B2	LL: A3Bx	15:02	15:27	11:01	11:02
**9**	E133F	EA	F	19.2	03:01	04:05	L: A3	L: A3	46:01	58:01	11:01	33:03
**10**	E133S1	EA	M	10	04:06	11:01	LL: A3Bx	L: B1	15:27	27:04	11:01	11:01

**Table 3:** A summary of HLA and RCCX haplotypes in the E94 family with multiplex SLE-mortality.

Code	HLA-DR	HLA-B	HLA-A	RCCX-C4	CYP21	TNX	C4B:C4A
**E94P**	DR15	B7	A2	LL: C4A-C4B	21A-21B	XA-XB	3:1
	DR7	B18	A30	**SS: C4Bx-C4Bx**	**21B-21B**	**XA-XB**	
**E94-S1**	DR15	B51	A24	LL: C4A3-C4B1	21A-21B	XA-XB	3:1
	DR7	B18	A30	**SS: C4Bx-C4Bx**	**21B-21B**	**XA-XB**	
**E94-S2**	DR15	B7	A2	LL: C4A3-C4B1	21A-21B	XA-XB	2:2
	DR15	B7	A26	LL: C4A3-C4B1	**21A-21A**	XA-XB	
E94-S3*	DR7	B18	A30	n.a.			
	DR15	B51	A24	n.a.			
**E94S2-N1**	DR7	B15	A2	LS: C4A3-C4B2	21A-21B	XA-XB	2:2
	DR15	B7	A26	LL: C4A3-C4B1	**21A-21A**	XA-XB	
**E94S2-N2**	DR15	B7	A2	LL: C4A3-C4B1	21A-21B	XA-XB	2:2
	DR15	B41	A30	LL: C4A3-C4B1	21A-21B	XA-XB	
**E94S3-N3**	DR4	B35	A1	LL: C4A3-C4B1	21A-21B	XA-XB	3:1
	DR7	B18	A30	**SS: C4Bx-C4Bx**	**21B-21B**	**XA-XB**	

*Blood or DNA sample not available; n.a., not available for RCCX/C4 studies;

C4Bx, mutant C4B gene with no protein product;

**21A-21A**, subjects with double pseudogenes of CYP21A in a bimodular RCCX haplotype are carriers of cytochrome P450 21-hydroxylase deficiency, a disease known as congenital adrenal hyperplasia.

In **Figures 3A–C**, it was notable that subject HC73 (middle *lanes*) contained four copies of *C4* genes (LL/LL) that all coded for C4A protein. No *C4B* gene and no C4B protein were detectable (*lane* 2, **Figures 3B, C**, respectively). This is a typical example to account for C4B protein deficiency because no *C4B* structural gene was present.

It is noticeable that the expression level of C4A protein in HC74 was similar to that of C4B (**Figure 3C**), although this subject had three copies *C4A* and one copy of *C4B* gene. Analyses of DNA sequences for exons 1-9 and for exons 33-41 did not reveal non-synonymous amino acid sequence changes. We also amplified and sequenced *C4A*-specific fragments spanning exons 10-26, and exons 26-35. However, those sequences did not reveal nonsense mutations in the coding regions of *C4A* genes in HC74.

### A Subject With a *C4B* Gene but No C4B Protein (MS630)

**Figure 4:**
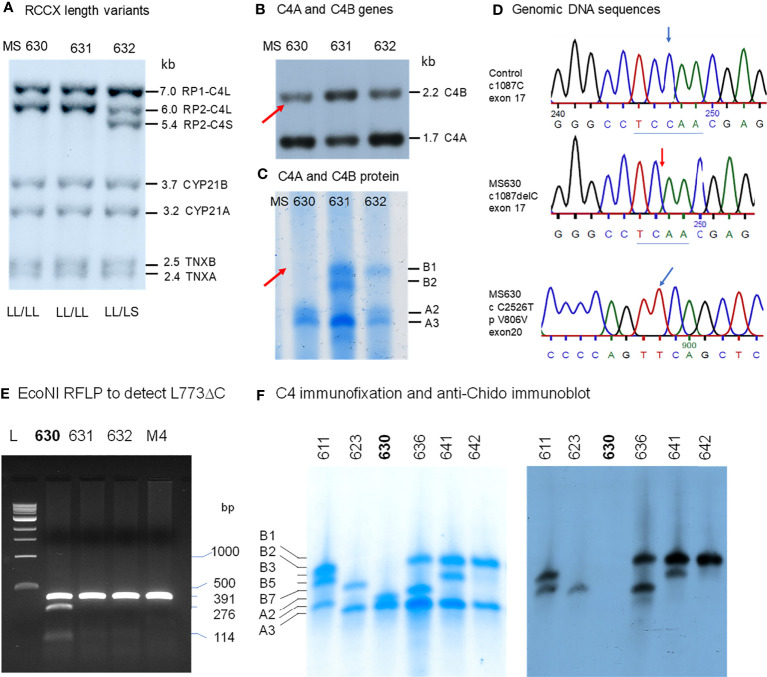
Molecular genetic analyses of a young European American subject with a mutant *C4B* gene (MS630). Experimental approaches were similar to those described in **Figure 3**. **(A)** *Taq*I RFLP of three consecutive subjects to show RCCX structures. **(B)** *Psh*AI/*Pvu*II to show the relative dosage of *C4A* and *C4B*. Notice that one *C4B* gene and three *C4A* genes were present in MS630 and MS632. **(C)** Immunofixation of C4A and C4B protein allotypes using EDTA-plasma from the same subjects shown in panels **(A, B)**. No C4B was detectable in MS630. **(D)** DNA sequences at exon 17 from a control subject (*upper panel*) and MS630 (*middle panel*) showing a single C-nucleotide deletion in codon 755 (Q755) of C4B in MS630. The *lower panel* shows a C→T polymorphism for codon 806 from exon 20 that did not change the amino acid sequence Val-806. **(E)** *Eco*NI RFLP to detect the C-nucleotide deletion for MS630 and control M4. A 391-bp DNA fragment spanning intron 16 (I-16.5) and the 5’ region of exon 18 (E18-r) was amplified by PCR, digested with *Eco*NI and resolved with agarose gel electrophoresis. *Panel* **(F)** shows results of immunofixation (left) and immunoblot (right) experiments for five subjects. C4 protein allotypes in MS630 did not react with anti-Chido monoclonal. The GenBank accession number for the DNA fragment containing sequence with C-deletion in MS630 is NZ203455.

In a young, healthy adult subject coded as MS630, genomic Southern blot analyses demonstrated the presence of four copies of *C4* genes in LL/LL configurations, as shown by *Taq*I RFLP (**Figure 4A**). There were three copies of *C4A* genes and one copy of *C4B* gene (**Figure 4B**). Those four *C4* genes coded for A3, A3 and A2 allotypes, but *no* C4B protein was detectable (**Figure 4C**). Immunoblot analyses using anti-Chido monoclonal did not detect any C4B-like protein in MS630 (**Figure 4F**). Thus, MS630 consisted of a copy of a mutant *C4B* gene that did not express a C4B protein.

To determine the molecular basis of mutation(s) leading to the absence of C4B protein production, the *C4B* gene in MS630 was amplified by long-range PCR in four DNA fragments similar to those described for the *C4B* gene in HC74 and sequenced. The output sequences were compared to those of healthy subjects. We discovered a deleterious C-nucleotide deletion at position 1087 of cDNA sequence for codon Qln-755 (Q755) from exon 17 (**Figure 4D**). This single nucleotide deletion changed the protein reading frame after Q755, abrogated the C1s cleavage site between Arg-Ala 756-757, and generated a nonsense TGA stop at codon 767 in exon 18 (Q755 ΔC fs12 767x). The C-deletion at codon Q755 also created a new cleavage site for restriction enzyme *Eco*NI.

To confirm the presence of the C-nucleotide deletion at codon Q755, genomic fragments of 391 bp with DNA sequences spanning between intron 16 and the 5’ region of exon 18 were amplified from four individuals including MS630, subjected to *Eco*NI digest and resolved by agarose gel electrophoresis. As shown in **Figure 4E**, new restriction fragments of 276-bp and 114-bp were found in MS630, in addition to the 391-bp fragment from regular *C4* genes (including *C4A* in MS630) without the ΔC at Q755. Genotyping of the HLA class II and class I genes revealed that MS630 had heterozygous DRB1*04:07 and *15:01; homozygous HLA-B*07:02; and heterozygous HLA-A*03:01 and *31:01 (**Table 2**).

We performed an epidemiologic study to screen for such Gln-755ΔC mutation in *C4* genes by *Eco*NI RFLP of PCR amplified samples. In a screening of >500 Black and White healthy subjects and patients with SLE, we did *not* detect a recurrence of this mutation.

### A Family With Multiplex SLE-Related Mortality and Heterozygous Deficiencies of C4A and C4B (E94)

#### Clinical Studies

**Figure 5:**
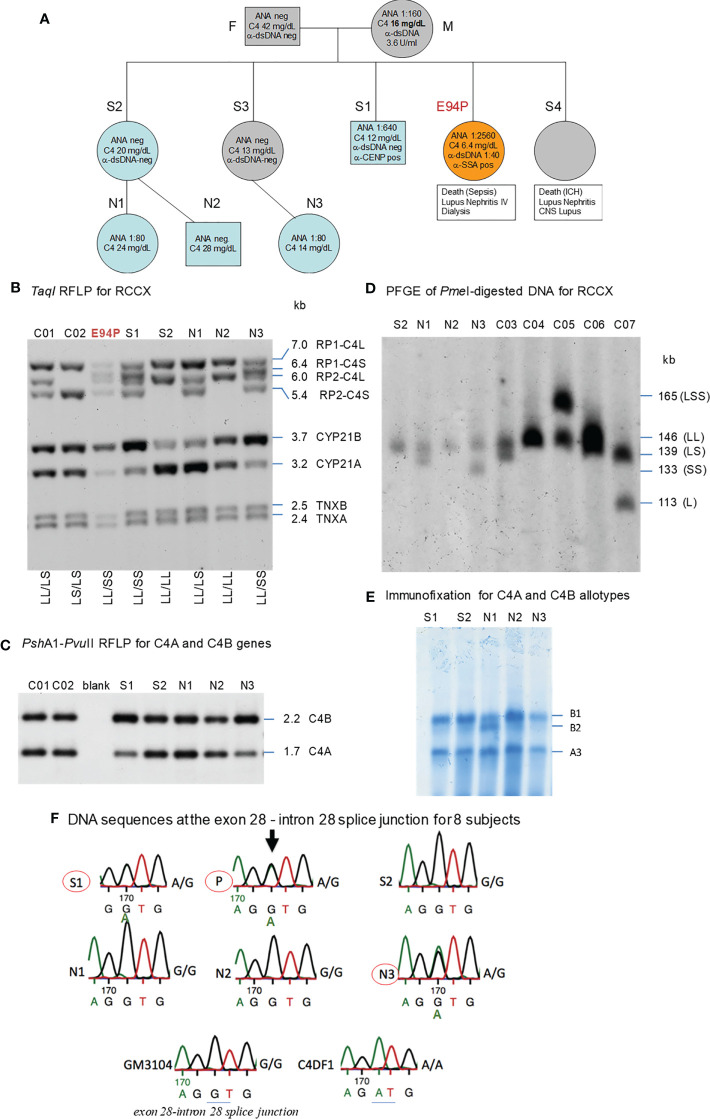
A European family with multiplex SLE-related mortality and low C4 levels (E94). **(A)** A three-generation family tree. E94P and S4 passed away with lupus nephritis and complications. **(B)** *Taq*I RFLP to show RCCX modular structures for six subjects of the E94 family including the index patient E94P, two siblings (S1 and S2), three niece/nephew plus two control subjects (C01 and C02). Note that E94P, S1 and N3 each contained an unusual short-short (*SS*) haplotype for RCCX that had *CYP21B-CYP21B* configurations (**Table 2**). **(C)** *Psh*AI/*Pvu*II RFLP to show the relative dosages of *C4A* and *C4B* genes for subjects shown in *panel* **(B)**, except E94P as her *C4A* and *C4B* genes were determined by real-time PCR. **(D)** Long-range mapping of the RCCX haplotypes by *Pme*I-digested genomic DNA in agarose plugs, resolved by pulsed-field gel electrophoresis and hybridized to radioactive probes specific for RCCX genes. DNA plugs for four members of the E94 family were prepared together with four unrelated controls (C03-C07). Subject N3 possessed the *LL* and *SS* haplotypes. **(E)** Immunofixation of EDTA-plasma to show C4A and C4B protein variants and their relative expression levels. Subject N3 has similar protein levels of C4A3 and C4B1, although she had *LL/SS* haplotypes with three copies of *C4B* gene and one copy of *C4A*. **(F)** DNA sequences at exon 28-intron 28 splice junction for six members of the E94 family, plus a C4 complete deficiency subject with identical HLA haplotypes (C4DF1), and a normal control (GN3104). Subjects S1, P and N3 had double sequences at the intron 28 splice junction donor site (marked by an *arrow* for E94P). C4DF1 has homozygous mutations at the same locations.

This multiplex SLE family came from Austria and Northern Italy. The index patient was a female (E94P) who was diagnosed with lupus at 35 years old and treated with azathioprine and steroids therapy. She developed nephritis with hematuria and proteinuria in the following several years. The main clinical laboratory results were as follows: anti-nuclear antibody (ANA) titer very high at 1:2,560; anti-double stranded DNA detectable at 1:40; anti-SSA (Ro) positive; serum C3 very low at 35.5 mg/dL; C4 very low at 6.4 mg/dL (**Figure 5A**). The patient contracted a fulminant pulmonary infection and died from therapy-refractory septic shock.

The family history of this patient was indicative of hereditary lupus nephritis. Her elder sister (E94-S4) developed severe lupus nephritis and lupus vasculitis at the age of 27. Despite intensified immunosuppressive therapy, her disease progressed to central nervous system vasculitis with intracranial hemorrhage, which became lethal (family pedigree shown in **Figure 5A**).

As two family members were diagnosed with renal SLE and died at young ages, we screened other family members - two other sisters, one brother, their parents, two nieces and a nephew for clinical signs of SLE. All other family members were asymptomatic of SLE at the time of recruitment for this study. The father (F) and one sister (S2) had normal clinical laboratory results. However, the mother (M), another sister (S3), the brother (S1), and one niece (N3) had low to borderline low serum C4 protein levels of 12-16 mg/dL (lower limit for healthy subjects: 15 mg/dL). The patient’s mother tested positive for anti-dsDNA (3.6 U/ml) and ANA (1:160). Her brother tested positive for ANA (1:640) and anti-CENP; and two nieces (N1 and N3) tested positive for ANA with relatively low titers (1:80). Limited quantity of genomic DNA from the index patient (E94P), plus blood samples from two of her siblings S1 and S2, two nieces N1 and N3 and a nephew N2 were available for studies of complement *C4* genetic diversity (**Figure 5A**).

#### RCCX and Complement *C4* Diversity

As shown in **Figure 5B**, *Taq*I genomic RFLP demonstrated that E94P and her niece N3 were both heterozygous for *LL* and *SS* with two long or two short *C4* genes in haplotypes, i.e., a total of four copies of *C4* genes. S2 and N2 had *LL/LL* haplotypes with four long *C4* genes; while N1 was heterozygous with LL/LS, three long and one short *C4* genes.

On examining the constituents of the RCCX modules, the patient (E94P), her sibling S1 and her niece N3 shared a distinct haplotype characterized by the presence of two short *C4* genes (*SS*) accompanied by two functional *CYP21B* genes (21B-21B; 3.7 kb; **Figure 5B**). In contrast, S2 and N1 each showed a bimodular *LL* haplotype containing two mutant *CYP21A* genes (*21A-21A*; 3.2 kb; **Figure 5B**). N2 had regular constituents with one pseudogene and one functional gene for CYP21-hydroxylase, i.e., *21A-21B* in each of his bimodular RCCX haplotypes.

**Figure 5C** with Southern blot of *Psh*AI-*Pvu*II RFLP showed that S2, N1, N2 each had equal copy numbers of *C4A* genes and *C4B* genes, which equated to two copies for each of them. S1 and N3 presented with three copies of *C4B* and one copy of *C4A*. *C4A* and *C4B* gene copy numbers of E94P were determined by quantitative real-time PCR (36), which yielded one copy of *C4A* and three copies of *C4B*. The combined results suggested that *C4* haplotypes of E94P, sister S1 and niece N3 were *C4A-C4B* and *C4B-C4B*.

Long range mapping using PFGE of *Pme*I digested DNA (**Figure 5D**) for four of the E94 family members confirmed the LL/LL structures for S2 and N2, heterozygous LL/LS for N1, and LL/SS for N3. Subjects C03 to C07 in **Figure 5D** were control samples with defined monomodular, bimodular and trimodular RCCX structures with different combinations of long and short genes (C03: *LL/LS*; C04: *LL/LL*; C05: *LSS/LL*; C06: *LL/LS*; C07: *LS/L*).

**Figure 5E** showed immunofixation results for C4 protein allotypes for five members of the E94 family. All members had C4A3 and C4B1. Subject N1 had an additional C4B2 allotype. Although subjects S1 and N3 each had *one* copy of *C4A* gene and *three* copies of *C4B* genes, the intra-individual protein band intensities for C4A3 and C4B1 allotypes in S1, and in N3, were similar. Such phenotypes suggested the possible mutations in two of the three *C4B* genes in S1 and N3, and likely the index patient E94P that reduced the expression of C4B protein to a level similar to that of C4A.

#### HLA Genotyping of the E94 Family

We investigated into the HLA class I and class II genotypes in this multiplex SLE family. HLA-A, B and DR alleles were ascertained by serology and/or DNA oligotyping. The availability of multiple family members for this study allowed segregations of HLA and RCCX haplotypes, which are shown in **Table 3**.

Notably, the index patient E94P had haplotype with *HLA-A30, B18* and *DR7* that segregated with *RCCX*: *SS*, *C4Bx-C4Bx*, *21B-21B*, *XA-XB*. This *A30-B18-DR7-SS* haplotype was shared with (a) her SLE sibling S3 who had CNS lupus and lupus nephritis and passed away of intracerebral hemorrhage, (b) another sibling S1 who had high titers of antinuclear autoantibodies (ANA, 1:640) and low level of serum C4 (12 mg/dL), and (c) niece N3 who had low level of serum C4 (14 mg/dL) and exhibited positivity of antinuclear antibodies. The other haplotype of E94P was *HLA-A2, B7* and *DR15* that segregated with *RCCX: LL*, *C4A-C4B*, *21A-21B* and *XA-XB*, which was also present in sibling S2 and niece N2. Subjects S2 and N2 were healthy at the time of recruitment.

The third HLA haplotype of interest in this E94 family was *A26, B7* and *DR15* that segregated with *RCCX: LL*, *C4A3-C4B1*, *21A-21A* and *XA-XB*. This is a carrier haplotype for cytochrome P450 21-hydroxylase (CYP21) deficiency that is one of the causes for congenital adrenal hyperplasia (CAH) (17). Such CAH-carrier haplotype was present in S2 and her daughter N1.

#### DNA Sequence Analyses of the *C4B* Genes in the E94 Family

The haplotype with HLA *A30 B18 DR7* and *RCCX: SS*, *C4Bx-C4Bx* and *21B-21B* is identical to two multiplex SLE families in Austria with complete C4 deficiency reported by us previously (33). The molecular basis of C4 deficiency for both *C4B* genes was a splice junction defect with T to A mutation at the donor site of intron 28 (GT→AT). Thus, DNA fragments spanning exon 28 and intron 28 of *C4* genes for six family members, E94P, S1, S2, N1, N2, N3, a completely C4-deficient patient (C4DF1) (33) who was homozygous with this haplotype, plus an unrelated control (GM3104) were amplified by PCR and sequenced by Sanger’s method. **Figure 5F** highlighted the sequence variations at the exon 28-intron 28 splice junction for those *eight* subjects. While GM3104 showed homozygous GT and C4DF1 homozygous AT sequences at the exon 28-intron 28 splice junction, three subjects including E94P, S1 and N3 had heterozygous GT/AT exon/intron 28 splice junction sequences. Three other subjects of the family, S2, N1 and N2 had the normal GT- sequences at the corresponding locations. Such results are consistent with the presence of the *HLA A30*, *B18*, *DR7*, *RCCX: SS*, *C4Bx-C4Bx*, *21B-21B* haplotype in E94P, S1 and N3.

### An East-Asian Subject With Anti-NMDA Receptor Encephalitis and C4B Deficiency

#### Molecular Basis of C4B-Deficieny in the E133 Family

**Figure 6:**
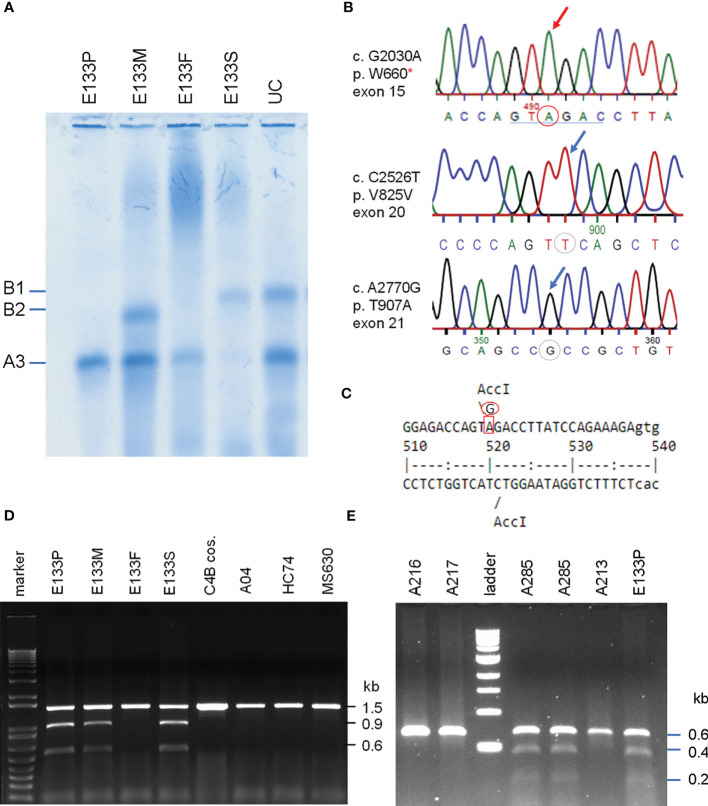
Identification of the molecular defect leading to C4B deficiency in an Asian patient with anti-NMDA receptor encephalitis (E133P). **(A)** Immunofixation of EDTA-plasma for C4 protein of the E133 patient (P), parents (M and F), and step-brother (S) plus an unrelated individual (UC). **(B)** Genomic DNA sequences showing conversion of W660 codon TGG conversion to nonsense codon TAG (W660x, upper). *Middle* and *lower* panels show two additional sequence variations for V825V from exon 20 (no change in amino acid) and for T907A from exon 21. **(C)** Genomic sequence that caused W660x mutation was distinguishable by *Acc*I RFLP. **(D)** Analyses of the E133 family and control subjects. An 1.5 kb DNA fragment spanning between exon 14 to exon 18 from eight subjects were amplified by PCR, digested with restriction enzyme *Acc*I and resolved by agarose gel electrophoresis. The results revealed that the W660x mutation was also present in his mother (E133M) and his stepbrother (E133S). **(E)** An improved method to detect W660x using a 0.6 kb fragment from intron 14 to intron 16 for *Acc*I RFLP. Subject A285 was performed in duplicate to confirm the presence of W660x. The molecular weight marker was the kb-ladder. The GenBank accession number for the DNA fragment containing sequence with W660x from E133P is NZ203456.

A fourteen-year-old boy was diagnosed with anti-NMDA receptor encephalitis as he presented with hypersomnolence, confused speech with echolalia, self-muttering, dysarthria, mood fluctuation, bilateral upper limbs tremor and headache (39). Immunological determination demonstrated detectable anti-NMDA receptor antibodies in the cerebrospinal fluid but not the serum. He had persistently low serum C4 concentrations (7 mg/dL at the time of recruitment). Treatment with intravenous immunoglobulin and pulsed methylprednisolone showed no effect at the beginning. However, after therapy with two sessions of plasmaphereses that replenished complement deficiency, and four doses of rituximab to suppress B-cell activity, this patient’s conditions improved remarkably. Our earlier case report showed that the patient had *RCCX* haplotypes *LL/L* with three copies of long *C4* genes (39). Of those *C4* genes were two copies of *C4A* and one copy of *C4B*. However, no C4B protein was detectable by immunofixation experiment (*lane* 1, **Figure 6A**) and thus patient E133P carried a mutant *C4B* gene. The patient’s father had homozygous, monomodular-long *RCCX* (*L/L*) haplotypes with two copies of *C4* genes both coding for C4A protein (lane 3, **Figure 6A**) and thus also had a homozygous C4B deficiency (39).

To investigate the molecular basis of C4B-deficiency in this encephalitis patient, coded E133P, genomic DNA for the coding regions of the long mutant *C4B* gene was amplified by long-range PCR as four separate DNA fragments, as described earlier for HC74 and MS630, and sequenced by Sanger’s method. Sequence analyses identified a G2030A change that knocked out the codon for tryptophan-660 residue to a nonsense stop codon (TGG→TAG, W660x) at exon 15 (**Figure 6B**). The DNA mutation for W660x created a new cleavage site for the *Acc*I restriction enzyme (GTAGAC, **Figure 6C**). Thus, DNA fragments of 1.5 kb spanning between exons 14 and exon 18 were amplified by PCR (using primer E14F and primer E18R) from patient E133P, his parents and a maternal stepbrother, a C4B cosmid and three unrelated subjects (A04, HC74 and MS630). The amplified DNA fragments were subjected to *Acc*I digest and resolved by agarose gel electrophoresis (**Figure 6D**). *Acc*I digested fragments of 0.9 kb and 0.6 kb were present in the patient (E133P), his mother (E133M) and maternal stepbrother (E133S), but not in his father (E133F) and four unrelated controls. Genotyping of the HLA class II gene *DRB1*, class I gene *HLA-B* and *HLA-A* were performed using genomic DNA from this E133 family (**Table 2**). Analyses of HLA haplotypes revealed that the *RCCX-LL* with *A3 and *C4B* mutation allele *Bx segregated with HLA-DRB1*04:06, HLA-B*15:27, HLA-A*11:01. The patient’s paternal haplotype with a single *C4A* gene segregate with HLA-DRB1*04:05, HLA-B*46:01 and HLA-A*11:01 (**Table 2**).

#### Screening of the W660x Mutation in *C4* Genes in Healthy Subjects and SLE

To facilitate screening of the W660x mutation, a more robust version *Acc*I RFLP analyses was developed. We reduced the amplicon size from 1.5 kb to 600-bp spanning from intron 14 (I-14) to intron 16 (I-16) of *C4* genes. This improved method was applied to screen healthy subjects and patients with SLE (11).

The W660x nonsense mutation was firstly detected in an independent East-Asian healthy subject, A-285, residing in the US (panel E, **Figure 6**). This subject had a total of four copies of *C4* genes organized in *LL/LL* structures for *RCCX*. Among those *C4* genes were two copies of *C4A* and two copies of *C4B*. The phenotypes of his C4 protein allotypes were A3, A3 and B1, with C4A3 in greater quantity than C4B1, which implied that one of the two *C4B* genes was not producing a C4B protein. Thus, A-285 likely had heterozygous *C4B* deficiency caused by the W660x.

Altogether we have screened >1000 subjects (700 healthy subjects and 320 SLE patients) for the presence of W660x mutation in *C4B* genes. Among the self-reported healthy subjects were 468 East-Asians, 118 European-Americans and 114 African Americans. All SLE patients screened were East-Asians (11). We identified 7 unrelated subjects who were positive for the W660x mutation among healthy East-Asians, a frequency of 1.5% in healthy subjects we screened. Unexpectedly, we did *not* detect the W660x mutation in anyone of the East-Asian SLE patient samples, or in non-Asian healthy subjects.

Genotyping of *HLA-DRB1*, *HLA-A, B* and *C* were performed on the seven W660x-positive subject. Remarkably, those seven subjects all shared common alleles with HLA-DRB1*04:06, HLA-B*15:27, HLA-C*04:01 and *LL* for *C4A3-C4Bx* in *RCCX* (**Table 4**). This haplotype with allelic combinations for *HLA-DRB1*, *HLA-B* and *RCCX-LL* is identical to that of the E133 family. Alleles for the more remote *HLA-A* (**Figure 1A**) exhibited diversity and three shared subtypes were found: *33:03, *24:02 and *11:01/02.

**Table 4:** HLA genotypes and RCCX modules in East-Asian subjects with W660x mutation in *C4B*.

Sample ID	DRB1_1	DRB1_2	RCCX-1	RCCX-2	C4B	C4A	C4T	HLA B_1	HLA B_2	HLA C_1	HLA C_2	HLA A_1	HLA A_2	C4 Protein
**1131**	**04:06**	03:01	na	na	1*	2	3	**15:27**	58:01	**04:01**	03:02	33:03	03:01	no C4B
**1710**	**04:06**	13:02	na	na	3	1	4	**15:27**	58:01	**04:01**	03:02	33:03	11:12	na
**1785**	**04:06**	07:01	** LL**	LS	2	2	4	**15:27**	57:01	**04:01**	06:02	11:02	01:01	na
**A-013**	**04:06**	09:01	** LL**	LL	2	2	4	**15:27**	40:06	**04:01**	03:04	24:02	26:01	C4A3>B1
**A-139**	**04:06**	08:03	** LL**	LS	2	2	4	**15:27**	46:01	**04:01**	01:02	24:02	02:07	C4A3>B2
**A-285**	**04:06**	12:02	** LL**	LL	2	2	4	**15:27**	46:01	**04:01**	01:02	11:01	26:01	C4A3>B1
**A-306**	**04:06**	15:01	** LL**	L	1*	2	3	**15:27**	15:12	**04:01**	04:03	11:01	02:03	no C4B

na, not available; shared HLA haplotypes are in bold. Homozygous C4B deficiency with a W660x mutation in one haplotype and an absence of a C4B gene in another haplotype is marked by an asterisk.

We looked into the C4 protein phenotypes in five of those seven subjects with W660x. Two of the W660x-positive subjects had single copy of *C4B* gene in a genome and thus manifested homozygous C4B protein deficiency, similar to the case in E133P. The other three subjects had reduced expression of C4B relative to C4A protein as expected.

## Discussion

Extensive protein polymorphism, frequent gene copy number variation, gene size dichotomy, and common genetic deficiencies of complement *C4A* or *C4B* are among the most striking features in the heritable human innate immunity and genetic diversities (9). Much attention has been focused on the roles for low copy numbers of total *C4* or *C4A* deficiency in autoimmune disease (12, 30, 43) and the overexpression of C4A in neurologic disorders (29). However, C4B protein allotypes are actually more polymorphic than C4A. C4B proteins possess greater chemical reactivities towards substrates with multiple hydroxyl groups such as carbohydrates, and have a higher frequency of genetic deficiency or the absence of *C4B* genes in most human populations (11, 12, 44–46). In this article, we presented interesting cases of fast migrating C4B polymorphisms, and elucidated molecular bases of *C4B* deficiency caused by genetic mutations rather than the absence of a structural gene.

We demonstrated previously that the *slowest* migrating C4B protein, C4B96, was caused by the E920K polymorphism that changed an acidic residue (Glu) to a basic residue (Lys) (11). Here we found that the novel sequence variation leading to the *fastest* migrating C4B in HC74, C4B7, is likely because of R729Q, a change from the basic Arg-729 to uncharged Gln-729, plus the conversion of the neutral Gly-1073 to acidic Asp-1073 (G1073D) that is usually present in C4A. Two other C4B allotypes, C4B2 and C4B5, which migrated faster than C4B1 but slower than C4B7 in an allotyping gel, also have Asp-1073 in their sequences (9).

*Nci*I RFLP of PCR-amplified DNA fragments enabled us to identify multiple subjects from a database with >1500 subjects with the polymorphism specific for R729Q in the anaphylatoxin domain of the C4B protein. Immunofixation and immunoblot analyses of plasma proteins confirmed those subjects produced the fast-migrating C4B7 protein and reacted with the anti-Chido monoclonal antibody that mostly recognized C4B.

The frequent gene copy number variation (CNV) of complement *C4* not only confers qualitative polymorphic protein variants and quantitative diversity with a large range of protein concentrations in plasma or serum samples, but also considerable isotype *deficiencies* of C4A or C4B. In a study of 517 *healthy* subjects of European ancestry in Ohio, homozygous deficiency of C4A protein due to the absence *C4A* genes in a diploid genome existed in 0.97% of the population, and homozygous deficiency of C4B due to the absence of *C4B* genes had a frequency of 2.71% (12, 17). Examples of homozygous C4B deficiency because of the absence of *C4B* genes shown here included HC73 (**Figure 3**) and E133F (**Figure 6**) (39). Heterozygous deficiency due to the presence of single copy of *C4A* in a diploid genome has a frequency of 17.2%, and single copy of *C4B* a frequency of 26.7%. These are very frequent genetic immunodeficiencies created by gene CNVs. On the other hand, deficiency of a C4A or a C4B protein due to nonsense sequence mutations or mini-indels are uncommon except for a 2-bp insertion into codon 1232 (formerly 1213) that is detectable in 1-2% of *C4A* genes in White patients with SLE (12).

Nonsense mutations specific for C4B deficiency had *not* been reported previously. Parallel genotyping and phenotyping experiments aided us identifying subjects who had *C4B* genes but produced no C4B proteins, unequivocally. In our first case of C4B deficiency with a mutant gene, a single C-nucleotide deletion (ΔC) in the coding sequence for Gln-755 (Q755), leading to frame shifts and generation of a stop codon at codon 767, was found in a young Caucasian with no clinical symptoms at the time of study. Screenings for >500 healthy subjects and autoimmune disease patients from multiple racial groups for this Q755-ΔC did not detect this mutation again. Such a phenomenon reaffirms that some deleterious genetic mutations are mostly private changes and scarcely recurrent in the general populations (24, 47, 48).

In a family residing in Austria and northern Italy with multiplex SLE mortality, nephritis, low C4 protein levels and the presence of antinuclear antibodies, we detected heterozygous C4 deficiency with two identical mutations with splice junction defects at the donor sites of intron 28 for both *C4B* mutant genes. Those mutant *C4B* genes are present in a bimodular short-short (*SS*) haplotype linked to *HLA A30, B18* and *DR7*. Intriguingly, such *HLA-A30 B18 DR7* haplotype with two identical *C4B* mutations at the splice junction of exon-intron 28 were found earlier in two other families with multiplex SLE nephritis. Those families also resided in Austria and northern Italy (33). Thus, deleterious mutations could be recurrent in families with multiplex disease located in nearby geographic locations of the index patients.

Anti-NMDA receptor encephalitis is a serious autoimmune disease mediated by antibodies against the NR1 subunit of the NMDA receptor, which reduces NMDARs surface density and synaptic localization (49–53). In the brain, complement C4 can be produced by, or deposited on, neurons and synapses which work with other components of the classical complement cascade to mediate synaptic pruning (29). The two human C4 isotypes C4A and C4B exhibited distinct relationships with synaptic pruning possibly caused by different affinities to unknown binding sites on synapses. High C4A seems to be a risk factor but C4B a protective factor on dysfunctional synaptic pruning (27, 29). Amplification and sequencing of a mutant *C4B* gene facilitated identification of W660x in an East-Asian patient with anti-NMDA receptor encephalitis. A specific *Acc*I-RFLP was created to identify this mutation. Genotyping and segregation analyses of the E133 family revealed that the mutant *C4B* gene was present in a haplotype with HLA-DRB1*04:06, RCCX-LL:C4*A3 *Bx, HLA-B*15:27 and HLA-A*11:01.

Application of the *Acc*I-RFLP enabled us to identify the W660x mutation in 7 unrelated healthy East-Asian subjects, with a prevalence frequency of 1.5%. Those seven subjects shared identical alleles with HLA-DRB1*04:06, HLA-B*15:27 and HLA-C*04:01 and RCCX-LL:C4*A3 *Bx. Strikingly, this mutation was *not* detectable in East Asian SLE, and in Black and White healthy subjects residing in Ohio. Such observation suggests that the W660x mutation is (a) likely specific to East-Asians, and (b) potentially protective against SLE.

Here we propose that complement C4A and C4B function like “*yin* and *yang”* physiologically. Activated C4A and C4B tend to interact or interfere with each other in moderating and propagating the activation of complement pathways (see **Supplementary Figure S2**), and in the fine-tuning of immunologic functions such as the induction of tolerance or suppression of autoimmunity. They are possibly engaged in the stimulation of type I interferon induced gene expression, in inflammatory response and complement-mediated tissue injuries (11, 30). While genetic deficiency or depletion of C4A is associated with systemic autoimmune diseases (11, 12, 30, 54), recent findings in neurologic disease suggested that high copy number of *C4A* or low copy number of *C4B* may be associated with schizophrenia (27, 28). In a cross-sectional study of human subjects with antiphospholipid (aPL) antibodies, those with median and high copy number of *C4B* (≥2) are strongly associated with thromboses and recurrent pregnancy loss (RPL) for female subjects. Notably, in that study cohort, subjects with aPL and homozygous *C4A* deficiency *all* experienced RPL, but those with homozygous *C4B* deficiency were protected from RPL (26). A similar phenomenon was observed in animals injected with human antiphospholipid antibodies – those with complement deficiency were protected from fetal resorption (55). In a longitudinal studies of pediatric lupus patients, it was found that patients with chronic hypertension persistently presented with higher serum protein levels of complement C4 and C3, and higher gene copy number of *C4B* (25).

Accumulation of sequence data from multiple polymorphic human *C4* genes reveals the presence of two clusters of genetic variations (**Supplementary Results**, **Table S1** and **Figure S1**). Historically, the first cluster was documented in the nineteen eighties (9, 56, 57). It is a 2.3 kb region from exons 24 to 29 coding for the C4d region with polymorphic variants that modulate the chemical reactivities of the thioester bond and antigenicities for the Rg/Ch blood groups (8, 9, 57). The second cluster of variations becomes conspicuous through this study. It has multiple variants at a 2.0 kb region between exons 15 and 21 that encodes the β-α chain junction, and the C1s cleavage site for the activation of the classical and the lectin pathways. Such activation process releases C4a and transforms C4b conformationally to enable covalent binding to substrates through the exposed thioester domain (22, 58, 59). Whether those variants in this second cluster would affect the C4 activation by C1s or MASP2, and the potential activity of C4a are the subject for further investigations. It is of interest to note that the “non-functional” sex-limited protein (Slp), a duplicated C4 homolog in mice, has changes at the C1s cleavage site that abrogated its classical pathway of complement activation (60, 61).

In summary, we have determined the molecular basis for the fastest migrating allotype of C4B and three cases of C4B protein deficiencies caused by sequence mutations. Their *RCCX* and *HLA* haplotypes have also been elucidated. While initially discovered in an encephalitis patient, the W660x mutation in *C4B* gene is modestly prevalent among East-Asians. Amelioration of activated C4B function could reduce complement-mediated inflammation and tissue injuries. Thus, it probably makes sense to observe that W660x mutation in C4B was *not* detectable in subjects with systemic lupus erythematosus. We anticipate such new knowledge would help advance our understanding of MHC and disease associations.

## Data Availability Statement

The datasets presented in this study can be found in online repositories. The names of the repository/repositories and accession number(s) can be found in the article/**Supplementary Material**.

## Ethics Statement

The studies involving human participants were reviewed and approved by Institutional Review Board of the Nationwide Children’s Hospital. Written informed consent to participate in this study was provided by the participants’ legal guardian/next of kin.

## Author Contributions

DZ, BZ, SL, YW, and CYY performed experiments. TA did the HLA typing. MR, GC, JD, FB-S, WP, JA, SB, RH, WJ, DB, SA, and YLL participated in human subject recruitment and clinical studies. All authors participated in project design, data interpretation, writing, and approval of the manuscript. CYY is responsible for data integrity and overall execution of the project.

## Funding

This work was supported by National of Institutes of Health grants R01 AR073311 (CYY and DB) and R21 AR070509 (CYY) from the National Institute of Arthritis, Musculoskeletal and Skin Diseases (NIAMS).

## Conflict of Interest

The authors declare that the research was conducted in the absence of any commercial or financial relationships that could be construed as a potential conflict of interest.

## Publisher’s Note

All claims expressed in this article are solely those of the authors and do not necessarily represent those of their affiliated organizations, or those of the publisher, the editors and the reviewers. Any product that may be evaluated in this article, or claim that may be made by its manufacturer, is not guaranteed or endorsed by the publisher.

## Abbreviations

CNV, copy number variation; HLA, human leukocyte antigen; MHC, major histocompatibility complex; min, minutes; NMDA receptor, N-methyl-D-aspartate receptor; RCCX: *RP-C4-CYP21-TNX* modules; RFLP, restriction fragment length polymorphism; RPL, recurrent pregnancy loss; s, second; SLE, systemic lupus erythematosus; TD, type 1 diabetes; W660x, tryptophan-660 to stop codon mutation in *C4B* gene.
