# Supplementary material for: Human Complement C4B Allotypes and Deficiencies in Selected Cases With Autoimmune Diseases
Source: Front Immunol. 2021 Oct 26;12:739430. doi: 10.3389/fimmu.2021.739430 (PMC8577214; doi:10.3389/fimmu.2021.739430)
Supplement: Supplementary file 1 [file DataSheet_1.docx]

*Annotated human short C4B gene sequences at locus I of the RCCX*

***RP1/STK19*** intron 3 . . . . . . . . .

-3000 atcccatggagagggcacagggccctggtaaatagtaatatgagggagagagacaggagggaaagagggaggagtgagagggtaaagagggggggagagg -2901

. . . . . . . . . . .

-2900 agggggaggaggaggaaggaaggagggggaggaggagggggggaggaagagggggaggaggatgaagaggaggaggaagaagaagggtatgagaggtgga -2801

-2799a (*Bgl*II RFLP) . . . . . . . . .

-2800 a*g*gatctgagcaagaggtaagacaggaagagaaatgctgtcctgggggtggaggttggtagagagtgagggtggggatggaccatgtctctcatctctgc -2701

. Exon 4 (*RP1*) . . . . . . . . .

-2700 ttgtagGTCCTCAAGGCCTGTGATGGCCGACCGTATGCTGGGGCAGTGCAGAAATTTCTAGCTTCAGTACTTCCAGCCTGTGGGGACCTTAGTTTCCAGC -2601

(112) V L K A C D G R P Y A G A V Q K F L A S V L P A C G D L S F Q Q 143

. . . . . . . . . . .

-2600 AGGACCAAATGACACAGACCTTTGGCTTCAGGGACTCAGAAATCACgtgagacttgtggaaccaaccaaagtcaggcatctggtgcttccctgcctccct -2501

D Q M T Q T F G F R D S E I T (158)

. . . . . . . -2425t . . .

-2500 ccagttccatccagcctgtcctcctgtttttttggtgaacctgccagaaaagctgccaaaaagctgactcttctt*g*ttaataaaatgacccaagtttgta -2401

. . . . . . . . . . .

-2400 ttcctccccacaagagaggaggcctatcttacctgggccttagaaagagccctgaaatagaattcagttcttggtggcttatcaaaagcacacaggggcc -2301

. -2291c. . . . . . . . . .

-2300 tggcaggaa*g*tgtaaaagcttgatgttaatcatactgggactaagaggatagagaatggtaggagctgggatacccctaaacattcacattaaaacaaaa -2201

. . . . . . . . . . .

-2200 aaaacccaaagctaaaaaacaactgggcaggagctaaataaaaatctaattttgagaggctgtatctggctcaggcctcctactttgtaacccatggaat -2101

. . . . . . . . . . .

-2100 atgtgaaagcatttgaaaaactatagcactgatctcacatgggcagacacactctcagagagatgtggtgggagccatggcgcagtctgcctaggcagtg -2001

. . . . . . -1938g . . . .

-2000 gcaggagcgcagaagactctgattcctctcctcggtcctaagaccgaatgtgtgtcaggaca*t*gtggtcagggaagagaagctatttaactgaaccagta -1901

. . . . . . . . . . .

-1900 atagtagcaggaaaagaaaaagtggagggagggcagtccaggtagggggcctggaacaagcaactgcaccaacagaggcagttggtgcgagcacagaacc -1801

. . . . . . . . . . .

-1800 accccaggctgggattttgttatccagtctctcttgcatggttgcccgtgtttctggagacttgtgtaaacattaatggatgaggaggagagatggttct -1701

. . . . . Exon 5 (*RP1*) . . . . .

-1700 cagagcccagccctcatctctgctggcttcccactgccctcagGCATCTGGTGAATGCTGGAGTCCTCACCGTCCGAGATGCTGGGAGCTGGTGGCTAGC -1601

(159) H L V N A G V L T V R D A G S W W L A 177

*. . .RP1*|*RP2* **Breakpoint of Gene Duplication** . . . . .

-1600 TGTGCCTGGAGCTGGGAGATTCAT**CAAG**TACTTTGTTAAAGgtatcccatctgcagctcaagcctgcagcccctcaccttttggtggctcctcaggcctc -1501

V P G A G R F I K Y F V K G (191)

. .(-1463 to -1465, deleted) --- Exon 6 . . . . .

-1500 taggccttattcacctttcccctttcctgtgccactt*ctc*ctctagGGCGCCAGGCTGTCCTTAGCATGGTCCGGAAGGCAAAGTACCGGGAACTGCTCC -1401

(192) R Q A V L S M V R K A K Y R E L L L 209

. . . . . . . . . . .

-1400 TATCAGAGCTCCTGGGCCGGCGGGCGCCTGTCGTGGTGCGGCTTGGCCTCACCTACCATGTGCACGACCTCATTGGGGCCCAGCTAGTGGACTGgtgagt -1301

S E L L G R R A P V V V R L G L T Y H V H D L I G A Q L V D C (240)

. . . . . . . . . . .

-1300 ctttccctggcctctggcagattatggagcaatgacccaaagtgggatttcctcccagctcatgcttagtttcctagtgaaggccagtggctctcattct -1201

. . . . . . . . . . Exon 7

-1200 tctctggaacccgggagcaccccttcccaagttctaagttctcctcacagcttgagcctaggcgtctggctccagccttgtctttctcctgcacagCATC -1101

(241) I

. . . . . . . . . . .

-1100 TCTACCACTTCAGGAACCCTCCTCCGCCTGCCAGAGACATGAAGATTCTGCTCATCATTGCTCAGCTCCTCAGAGTGGGCCGGGAGGGGACTAGAAGAGC -1001

S T T S G T L L R L P E T * (254)

. . . . . . . . . . .

-1000 TGCATGATGGTGGCTGAGACAGGGTCACCTTGGGAAGGCTTGGGAGCCAGGATGAGTGTCGGGCTCTCGTGTGTGCAAAAGGTCAGATGTGACTGCTGCT -901

. . . . . . . . . . .

-900 GTTTGCCTGGTTTCTGACCCAGTGGTGGGGTTTGAGCAATGCTTCTCTGCCCTTCCATGGAAAGTGGAACCAGAAATGGTGCCAAGGCTGTGGCTGTTCC -801

. . . . . . . . . . .

-800 CTTTCGTGTAAAATGGTGCTGTTATTACTCTGTCTTGAAATAGGAAGGTGGGATTTCTGGGGAGGCTGGTGAAGGAGGGCAGGGTTCTTTTCTCTACGTG -701

. . poly(A) signal poly(A) site (RP1/STK19 . . . .

-700 TCATGTTAAAATTGCCA**AATAAA**GTACCTCTGCCTGTG**A**tattttctggatgtcctttatttactgtgacgtgtgtttgggtgccttgtttaggggtaga -601

. . . . . . . . . . .

-600 ggtgaagtctgagctttgcctcattcagagaggaaaggggtcaggggttcactctgacgttcaggccattctccctgtggagtggtgagggtgtacctaa -501

. . . . . . . . . . .

-500 tctcctaaaccacggaatttctgttagggcctaaaaaagcaaaagcctagtatagttcaatttgtgttggaatgaaagtaagagacaagtgtcttagaag -401

. . . . . . . . . . .

-400 cctgtcattgttttgtgagggcctttaaatatcctgtactcgtgggccatgttgggcccttgtacgcccaggtatacatgagcttgtgtgcacctatacc -301

. . . . . . . . . .γ-IFN IRE

-300 ctgatacagatatacctggtagggggaggtgctcaggcactggaatgagaggagttaacggggaaggacagggttatttctgggccaagattcagagttt -201

. CACCC Box . . . NF1 . . .E-Box Enhancer Core. SP1 .

-200 cccatggacacccaggtgtccggggtgcccccacaactctgggcctgaggccagttgcacttcttggctgt*cacgtg*gtttcccagcttagctgggctgg -101

. . . . . * Exon 1 **(*Complement C4***) . *Bam*HI .

-100 gggaggagcaaggtccagagtcaactctgccccgaggcctagcttggcc**A**GAAGGTAGCAGACAGACAGACGGATCTAACCTCTCTTGGATCCTCCAGCC -1

. . . . . . . . . . .

1 **ATG**AGGCTGCTCTGGGGGCTGATCTGGGCATCCAGCTTCTTCACCTTATCTCTGCAGAAGCCCAGgtcctggaggcgggatgctgggtgcttggattggg 100

(1) M R L L W G L I W A S S F F T L S L Q **K** P R (22**)**

**C4 Leader peptide | C4 N-terminus; MG1**:20-137

. . 116a (*Taq*I RFLP) . . . . . . . . Exon 2

101 gcagggctggcatcg*g*gacccgattcaggagtgagggagagcaggggtggaggtgtcagagcgaagtctgactgctgatcctgtctgttctccccagGTT 200

(23)L

*. . . . . . . . . .*

201 GCTCTTGTTCTCTCCTTCTGTGGTTCATCTGGGGGTCCCCCTATCGGTGGGGGTGCAGCTCCAGGATGTGCCCCGAGGACAGGTAGTGAAAGGATCAGTG 300

L L F S P S V V H L G V P L S V G V Q L Q D V P R G Q V V K G S V 56

. . . . . . . T378 . .

301 TTCCTGAGAAACCCATCTCGTAATAATGTCCCCTGCTCCCCAAAGGTGGACTTCACCCTTAGCTCAGAAAGAGACTT*C*GCACTCCTCAGTCTCCAGgtaa 400

F L R N P S R N N V P C S P K V D F T L S S E R D F A L L S L Q (88)

F82

. . . . . . . . . .

401 ccagaccccatgccctcctgctgcttgtgggggcctcctgccctgttcccatctgtcttgtaagtgtcatcatcttcccactggcctcctcccctcctgt 500

. . . . . . . . . .

501 cttcccaccctggcattctccttccacgtttctcccttggtctctgtcctttttggtcagctgtctcttgctctgtgacccgctccctctccctctccct 600

.Exon 3 . . . . . . . . .

601 ctcctgacagGTGCCCTTGAAAGATGCGAAGAGCTGTGGCCTCCATCAACTCCTCAGAGGCCCTGAGGTCCAGCTGGTGGCCCATTCGCCATGGCTAAAG 700

(89) V P L K D A K S C G L H Q L L R G P E V Q L V A H S P W L K 118

. . . . . . . . . .

701 GACTCTCTGTCCAGAACGACAAACATCCAGGGTATCAACCTGCTCTTCTCCTCTCGCCGGGGGCACCTCTTTTTGCAGACGGACCAGCCCATTTACAACC 800

D S L S R T T N I Q G I N L L F S S R R G H L F L Q T D Q P I Y N P 152

**MG2:**138-240

. . . . . . . . . .

801 CTGGCCAGCGGGgtgagtctcagccccagggcctcaacctttaaccccctccgagccctctcaggatgagtttggtgccccctaagtgagataacctgaa 900

G Q R V (156)

. . . . . . . . . .

901 agaaagtgccacacagaaggggtgcttaggaaacatttgtcccctgctccctctgtggagtttgacccaccctccccttgcacatggacccctgctcacc 1000

. . Exon 4 . . . . . . . .

1001 tctctcctcctccactcccagTTCGGTACCGGGTCTTTGCTCTGGATCAGAAGATGCGCCCGAGCACTGACACCATCACAGTCATGGTGGAGgtgagtcc 1100

(157) R Y R V F A L D Q K M R P S T D T I T V M V E (179)

. . . . . . .Exon 5 . . .

1101 ccgacctctggccttcctgatcctggccactgatgtgacctcctgcctgtgagcacttctccccttgcagAACTCTCACGGCCTCCGCGTGCGGAAGAAG 1200

(180) N S H G L R V R K K 170

. . . . . . . . . .

1201 GAGGTGTACATGCCCTCGTCCATCTTCCAGGATGACTTTGTGATCCCAGACATCTCAGAgtgagcgctcccaatgtgggggctgcccccaagctacacca 1300

E V Y M P S S I F Q D D F V I P D I S E (208)

. . . . . . . . . .

1301 ccccaattcctgttaggctctccacctcccacacagaggcacgtccccagatgccctgaccctcagcctcctgagcctctggttaacccccacagtcctc 1400

. . . . . . . Exon 6 . .

1401 ttcccagggaagcaggctgctggctctccgtgccccactgtacagatgggctgagccccttccttgtccattctcagGCCAGGGACCTGGAAGATCTCAG 1500

(210) P G T W K I S A 217

. . . . . . . . . .

1501 CCCGATTCTCAGATGGCCTGGAATCCAACAGCAGCACCCAGTTTGAGGTGAAGAAATATGgtgagagctggaaactggagggacaggcagctgctttcct 1600

R F S D G L E S **N** S S T Q F E V K K Y V (237)

N226 (Glycosylation-high mannose type)

. . . . . . . . . .

1601 gaaggaaataagggtggaaggagaggtactgggagcagctcagggcagggagatatgggtgccacagccctgagcagaggggagtctttgagctggagtc 1700

. . .Exon 7 . . . . . . .

1701 tgacctgcctatcccttcaccctgggtcagTCCTTCCCAACTTTGAGGTGAAGATCACCCCTGGAAAGCCCTACATCCTGACGGTGCCAGGCCATCTTGA 1800

(238) L P N F E V K I T P G K P Y I L T V P G H L D 260

**MG3**:241-364

. . . . . . . . . .

1801 TGAAATGCAGTTAGACATCCAGGCCAGgtaatacctccctccccacctctgcccaccagcaccgggtcctgctccctactcagtatgaatgggctcctgc 1900

E M Q L D I Q A R (269)

. . . . . . Exon 8 . . .

1901 TTCCCTGccctcgggccattattccccccagcccttggcccaccctcttctctctgccacgacagGTACATCTATGGGAAGCCAGTGCAGGGGGTGGCAT 2000

(270) Y I Y G K P V Q G V A Y 281

. . . . . . . . . .

2001 ATGTGCGCTTTGGGCTCCTAGATGAGGATGGTAAGAAGACTTTCTTTCGGGGGCTGGAGAGTCAGACCAAGgtaggaaggagaataggggctggggaggg 2100

V R F G L L D E D G K K T F F R G L E S Q T K (304)

. . . . . . Exon 9 . . .

2101 GAAGGGGcaagggaggtgaggtgggagactcagtctcaccctatgtcctgtttctttctatgccccagCTGGTGAATGGACAGAGCCACATTTCCCTCTC 2200

(305) L V N G Q S H I S L S 315

. . . . . . . . . A2296

2201 AAAGGCAGAGTTCCAGGACGCCCTGGAGAAGCTGAATATGGGCATTACTGACCTCCAGGGGCTGCGCCTCTACGTTGCTGCAGCCATCATTGAGT***C***TCCA 2300

K A E F Q D A L E K L N M G I T D L Q G L R L Y V A A A I I E **S** P 348

**Y**347

. . . . . . .a2371 . . .

2301 Ggtgggtgactttcccttattgtaaccccagacccttgcctctgacctctgagctaaccctctgtcctcc*g*gcaccaacaccaccccacttctcacatct 2400

G (349)

. . . . . . . . . .

2401 catctcagactcaaaaccaggaaacacccaggagacctggtttctctccaactctgtctctgtgactcggcccttttccctggctgagtttatttatttc 2500

. . . . . c2558 a2564 . c2577 . g2592 .

2501 tttgctcgttctgctcattccttcactcctccagtggacatgtgttgttcaatgccc*t*gtgct*g*ggcctcagcatg*t*a**cagaca**ggaatac*a*gcagcctg 2600

*HERV-K(C4) integration site for long C4 gene* (+6367 nt)|

t2601 . . . . .c2651 . . . . .

2601 *c*gccctgggagctcactgtcttgtgggagggaaccactcaagccactccc*t*acttgtcctcctgtccctctcttcttgggctctgtcccccacctctctc 2700

. Exon 10 . . . . . . . .

2701 tgtcctttgtcttgcagGTGGGGAGATGGAGGAGGCAGAGCTCACATCCTGGTATTTTGTGTCATCTCCCTTCTCCTTGGATCTTAGCAAGACCAAGCGA 2800

(350) G E M E E A E L T S W Y F V S S P F S L D L S K T K R 376

**MG4**:365-465

. . . . . . . . . .

2801 CACCTTGTGCCTGGGGCCCCCTTCCTGCTGCAGgtttcttccagaggggaaggatgagtagggaggatgtggtagttaggagggctcagggtctgaccac 2900

H L V P G A P F L L Q (387)

. . . Exon 11. . . . . . .

2901 tctcttttgcctgccctcctttacctgcctagGCCTTGGTCCGTGAGATGTCAGGCTCCCCAGCTTCTGGCATTCCTGTCAAAGTTTCTGCCACGGTGTC 3000

(369) A L V R E M S G S P A S G I P V K V S A T V S 410

. . C3024. . . . . . . .

3001 TTCTCCTGGGTCTGTTCCTGAAG*T*CCAGGACATTCAGCAAAACACAGACGGGAGCGGCCAAGTCAGCATTCCAATAATTATCCCTCAGACCATCTCAGAG 3100

S P G S V P E **V** Q D I Q Q N T D G S G Q V S I P I I I P Q T I S E 443

**A**418

. t3119 c3127 . . . . . . .

3101 CTGCAGCTCTCAgtagga*c*tcctcgg*a*cccctgggagatggtgggggaaggggaggagggtgagctggggtcccaaggatccatggcctgacttgggggg 3200

L Q L S (447)

. . . . . Exon 12 . . . .

3201 AAGGTGGggtacttggctctgagctactaccctattcgcacctgaccccctctccagGTATCTGCAGGCTCCCCACATCCAGCGATAGCCAGGCTCACTG 3300

(448) V S A G S P H P A I A R L T V 462

. . *Eag*I/*Eae*I RFLP 3345T T3349 *Taq*I . . . .

3301 TGGCAGCCCCACCTTCAGGAGGCCCCGGGTTTCTGTCTATTGAG*C*GGC*C*GGATTCTCGACCTCCTCGTGTTGGGGACACTCTGAACCTGAACTTGCGAGC 3400

A A P P S G G P G F L S I E **R P** D S R P P R V G D T L N L N L R A 495

**MG5**:466-563 (C4A6)477 **W** **L** 478 (C4B1-hemolytically inactive) C5 binding site

. . . . . 3463a . . . .

3401 CGTGGGCAGTGGGGCCACCTTTTCTCATTACTACTACATGgtgtgcatgagctggggagtca*c*ggagggctggggtgcagggaagagccctctgggtggg 3500

V G S G A T F S H Y Y Y M (508)

. . . . . . *C4A-long* *HLA A24 B38 DR13*, **ΔGT** 3594

3501 GCTGGGGgggttcaaggctgaggctgtcccatgaagaggcaaccactcttgtccctcccattcttggcccagATCCTATCCCGAGGGCAGATCGTGTTCA 3600

Exon 13 (509) I L S R G Q I V F M 518

V516 fs110, **626x**

. . . 1-bp del, C4BQ0, *HLA A2 B12 DR6*, 3671 **ΔC** 541F fs48, **588x**  **. .**

3601 TGAATCGAGAGCCCAAGAGGACCCTGACCTCGGTCTCGGTGTTTGTGGACCATCACCTGGCACCCTCCTT***C***TACTTTGTGGCCTTCTACTACCATGGAGA 3700

N R E P K R T L T S V S V F V D H H L A P S F Y F V A F Y Y H G D 551

. . **T**3723 C4A-mutation, *HLA A2 B17 DR7* . . . . .

3701 CCACCCAGTGGCCAACTCCCTG**C**GAGTGGATGTCCAGGCTGGGGCCTGCGAGGGCAAGgtgaccggggtcaggagagatggcacttgtgccgagggggtt 3800

H P V A N S L **R** V D V Q A G A C E G K (570)

**R559x** **MG6β**: 564-605

. . . . . . . . . .

3801 gaggacagggtgattgccaacagggcatggatttagcttgggggcagtgaggataccgggactgaaggaagctctcccactctgaccgcccccacctgcc 3900

. Exon 14 . . . . . . . . .

3901 gcccctgccagCTGGAGCTCAGCGTGGACGGTGCCAAGCAGTACCGGAACGGGGAGTCCGTGAAGCTCCACTTAGAAACCGACTCCCTAGCCCTGGTGGC 4000

(571) L E L S V D G A K Q Y R N G E S V K L H L E T D S L A L V A 600

. . . . . . . . . .

4001 GCTGGGAGCCTTGGACACAGCTCTGTATGCTGCAGGCAGCAAGTCCCACAAGCCCCTCAACATGGGCAAGgtttgtccagaccctctccacagctctctc 4100

L G A L D T A L Y A A G S K S H K P L N M G K (623)

**Link**:606-675

. . . . . . . . . .

4101 acccctccatggctcatccccctgcttccctgagccttgggcgcagcccctggatcccactgaggctccccacagtctcttccccacttggccctgtggt 4200

. . . . . . . . . .

4201 ctccatctcctggctctgtatcctttcctatccccccatgtgctgccctctcacctgtgccgagtgctcagtcctgcccctcagccacacttggctccta 4300

. . Exon 15 . . . . . . . .

4301 gcattcctgcctttcttgcagGTCTTTGAAGCTATGAACAGCTATGACCTCGGCTGTGGTCCTGGGGGTGGGGACAGTGCCCTTCAGGTGTTCCAGGCAG 4400

(624) V F E A M N S Y D L G **C**  G P G G G D S A L Q V F Q A A 650

S635 (protein sequencing)

. . .**A**4431, 660* E133 *Acc*I RFLP . . . . .

4401 CGGGCCTGGCCTTTTCTGATGGAGACCAG**TG*G***ACCTTATCCAGAAAGAgtgagaacagagaaggaaggggagtgggtggcgggaagataaggaaggagga 4500

G L A F S D G D Q **W** T L S R K R (666)

**W660x**

. . . . . . . . . .

4501 agggcctgaggggaccagctggaagagtccgggcaggaagggctgggcaggggaaggggaggaggggaggaggccgagtgcctgacggctggactgcagc 4600

. Exon 16 . . . . . . . .

4601 ctttctctctaccagGACTAAGCTGTCCCAAGGAGAAGACAACCCGGAAAAAGAGAAACGTGAACTTCCAAAAGGCGATTAATGAGAAATgtgagttgcg 4700

(667) L S C P K E K T T **R K K R** **N** V N F Q K A I N E K L (691)

**β chain ends** 🡨675| |680🡪 **α chain, C4a starts**

**ANA**:680-756

. . . . . . . . . .

4701 ggtgcctaggcagtagcttgggctctccacctgggatccgggttgggggtctgcctctctgcccctcggctccttgctgaacccacgtgtggtatttggg 4800

. . . . . . . . . .

4801 gccagagatccgaattccgggattacgagtggaaggtgggcagctctctccagcagcctctcttatgttgctggtctcaaggggtcggggcgggggctga 4900

. . . . Exon 17 . . . . .

4901 ggtgtatgtcctttttgtcctctcatgctcacccccacctggccctgcagTGGGTCAGTATGCTTCCCCGACAGCCAAGCGCTGCTGCCAGGATGGGGTG 5000

(692) G Q Y A S P T A K R C C Q D G V 707

. . . . (*Pvu*II RFLP) T5056 A5065. . . .

5001 ACACGTCTGCCCATGATGCGTTCCTGCGAGCAGCGGGCAGCCCGCGTGCAGCAGC*C*GGACTGCC***G***GGAGCCCTTCCTGTCCTGCTGCCAATTTGCTGAGA 5100

T R L P M M R S C E Q R A A R V Q Q **P** D C **R** *E* P F L S C C Q F A E S 741

**L**726 **Q**729 (C4B7, HC74)

. . . . **ΔC** Q755 fs12, 767* MS630 (*Eco*NI RFLP) . . .

5101 GTCTGCGCAAGAAGAGCAGGGACAAGGGCCAGGCGGGCCTC**C**AACGAGgtgaggggctgggtggggctagggcacaggtggcggcgcttggaaaggcaga 5200

L R K K S R D K G Q A G L **Q** R A (757)

D748-I760: R (sessile) loop | **αNT**:757-778

C1s cleavage site: **C4a ends**

***. . .*** Exon 18 ***. . . . . .***

5201 acggtcccctcctcactcccgtccaccgtggtcccccagCCCTGGAGATCCTGCAGGAGGAGGACCTGATTGATGAGGATGACATTCCCGTGCGCAGCTT 5300

(758) L E *I* L Q E E D L I D E D D I P V R S F 777

. . . . . . . . . .

5301 CTTCCCAGAGAACTGGCTCTGGAGAGTGGAAACAGTGGACCGCTTTCAAATgtgagagtgtgtgccggcccggccttttctctgtgctgtgtctcggggc 5400

F P E N W L W R V E T V D R F Q I (794)

**MG6α**:779-832

. . . . Exon 19 . . . . . .

5401 cagccggggtagacgggccttctctgcctttccctacacagATTGACACTGTGGCTCCCCGACTCTCTGACCACGTGGGAGATCCATGGCCTGAGCCTGT 5500

(795) L T L W L P D S L T T W E I H G L S L S 814

. . . . . . . . . .

5501 CCAAAACCAAAGgtgatgtcaccctgtctgggcctcaggtgaccctgcttccatttccctgtaccccagctccctgttccctttgctcttagtgtaggaa 5600

K T K G (818)

. . . . . . . . . .

5601 gagggtccagtgatctggggaggtctgtgccagcgtgcagctggcgtgggccagagggcagaggcggactgagacagagctgggtcacccccacccctcc 5700

. . . . . . .Exon 20 . . C5793 .

5701 ctcctgtggccctgaagctttgatggcccctctgatctctgcccctgtgcccacgcttcctttccctcagGCCTATGTGTGGCCACCCCAGT*T*CAGCTCC 5800

(819) L C V A T P **V** Q L R 828

**V**825

**ΔC** 5809 C4AQ0,R831 fs32 **863x**, *HLA A30 B18 DR3* . . . . .

5801 GGGTGTTC**C**GCGAGTTCCACCTGCACCTCCGCCTGCCCATGTCTGTCCGCCGCTTTGAGCAGCTGGAGCTGCGGCCTGTCCTCTATAACTACCTGGATAA 5900

V F **R**  E F H L H L R L P M S V R R F E Q L E L R P V L Y N Y L D K 861

**MG7**:833-935

863 . . a5923 . . . . . . . .

5901 AAACCTGACTgtgaggccccat*g*ggagcctgagcatacaggagttgggggagccagggcccagtgaggggtggggaggctaaccgggccaggactctggc 6000

N L T (864)

N862 (glycosylation-BAC)

. . Exon 21 . . . . T6077 . .

6001 catcctcgttttcctgccctcagGTGAGCGTCCACGTGTCCCCAGTGGAGGGGCTGTGCCTGGCTGGGGGCGGAGG*G*CTGGCCCAGCAGGTGCTGGTGCC 6100

(865) V S V H V S P V E G L C L A G G G G L A Q Q V L V P 890

G882

. . . . G6150 . . . A6189 *Eco*RI RFLP

6101 TGCGGGCTCTGCCCGGCCTGTTGCCTTCTCTGTGGTGCCCACGGCAGCC*A*CCGCTGTGTCTCTGAAGGTGGTGGCTCGAGGGTCCTTC*G*AATTCCCTGTG 6200

A G S A R P V A F S V V P T A A **T** A V S L K V V A R G S F **E** F P V 923

**A**907 **K**920 (C4B96)

. . . . . . . t6272 . . .

6201 GGAGATGCGGTGTCCAAGGTTCTGCAGATTGAGgtgaatggagcacccctgaatataagtccccgggcccc*c*agctttgtcctccaccctcagcactctc 6300

G D A V S K V L Q I E (934)

. . .g6331 . . . . . . .

6301 tctgctggccaggccaggggcccaacaccc*a*aaccaatgccttggtctgttcccatcttctacaattctgatccaactctgtccctggagttgaaactca 6400

. c6419 . . . . . Exon 22 . .

6401 aagttctgggggagtctg*t*gctagcagggcaggctgtagtcctgtgtgacctcacaaccatgttttccctgagacagAAGGAAGGGGCCATCCATAGAGA 6500

(935) K E G A I H R E 942

**CUBf**:936-983

. . . . . . . . . .

6501 GGAGCTGGTCTATGAACTCAACCCCTTGGgtgagtgaccctctacctccagccattggtttcctaagtgggtacaggtggtgggggatgtggacagcagg 6600

E L V Y E L N P L D (952)

. . .Exon 23 . . . . . . .

6601 acaggctgccaacttcccccatttccccagACCACCGAGGCCGGACCTTGGAAATACCTGGCAACTCTGATCCCAATATGATCCCTGATGGGGACTTTAA 6700

(953) H R G **R** T L E I P G N S D P N M I P D G D F N 975

factor I cleavage site 956 | 957🡪 **C4d starts**

. . . . . . . t6775. . .

6701 CAGCTACGTCAGGGTTACAGgtgggagtgccctttagtcccttcccagtggccaccttcggattcatgtgggac*c*tgtggatccctgcttggtcccactc 6800

S Y V R V T A (982)

. . . . . Exon 24 . . . .

6801 CCCGTGAgcctctgacacagagtcctcagacctccaccctctccctcccatgtagCCTCAGATCCATTGGACACTTTAGGCTCTGAGGGGGCCTTGTCAC 6900

(983) S D P L D T L G S E G A L S P 998

**TED**:984-1323

. . . . . . . . . .

6901 CAGGAGGCGTGGCCTCCCTCTTGAGGCTTCCTCGAGGCTGTGGGGAGCAAACCATGATCTACTTGGCTCCGACACTGGCTGCTTCCCGCTACCTGGACAA 7000

G G V A S L L R L P R G **C G E Q** T M I Y L A P T L A A S R Y L D K 1030

1010-thioester-1013

. 7022T . . . . . . . .

7001 GACAGAGCAGTGGAGCACACT*G*CCTCCCGAGACCAAGGACCACGCCGTGGATCTGATCCAGAAAGgttctgggtgcaagggcaagcaggaggggggccag 7100

T E Q W S T **L** P P E T K D H A V D L I Q K G (1052)

L1037

. . . . . . . . . .

7101 gaaaggacagttactggaagatggacagcccaggaggctacagagggaaagaaagggggcccctgatgaggatggggagcatggccttgggctcaaacag 7200

. . . . Exon 25 . . . 7292T .

7201 cagaagggtgagtgtcacctgagcggccacctctcctctccaagGCTACATGCGGATCCAGCAGTTTCGGAAGGCGGATGGTTCCTATGCG*G*CTTGGTTG 7300

(1053) Y M R I Q Q F R K A D G S Y A **A** W L 1070

**V**1068

A7308 T7318 . . . . . . . .

7301 TCACGGG*G*CAGCAGCAC*C*TGgtgagcttgggagagtggttccagggttctgagggggtcagggctggggcaggggtgggacagagctggtatgatgggag 7400

S R **G** S S **T** W (1077)

(*Ch5*) **D**1073 T1076

. . . . . . . .Exon 26 . .

7401 ggtggataaccaggcacctgggggcgtgggcataatgagaagcaagtccttatccccaaccctcctttcctgccctccagGCTCACAGCCTTTGTGTTGA 7500

(1078) L T A F V L K 1084

. . . A7335. . . . . . .

7501 AGGTCCTGAGTTTGGCCCAGGAGCAGGTAGGAGG*C*TCGCCTGAGAAACTGCAGGAGACATCTAACTGGCTTCTGTCCCAGCAGCAGGCTGACGGCTCGTT 7600

V L S L A Q E Q V G G S P E K L Q E T S N W L L **S**  Q Q Q A D G S F 1117

G1095 **I**1109 (protein sequencing)

*Psh*AI RFLP (C4A)

7609C. G T G C7625. . . . . . . .

7601 CCAGGACC**T**CT**C**TCCAGTG**A**TA**C**A**T**AGGAGCATGCAGgtgcgggcatgctggggctggcccgagaagcgcctgtcggaggactctctttgccccttcccc 7700

Q D **L** **S** P V **I** **H** R S M Q (1129)

1120**P** **C** **L** **D**1125 (*C4B/C4A isotypic residues*)

. . . Exon 27. . . . . . .

7701 ctcctgtttgacatcttttctccccttactagGGGGGTTTGGTGGGCAATGATGAGACTGTGGCACTCACAGCCTTTGTGACCATCGCCCTTCATCATGG 7800

(1130) G G L V G N D E T V A L T A F V T I A L H H G 1152

. . . . . . . . . .

7801 GCTGGCCGTCTTCCAGGATGAGGGTGCAGAGCCATTGAAGCAGAGAGTGgtaagttcagtggcgtttctgccctctgctggcccccagctctctcccttt 7900

L A V F Q D E G A E P L K Q R V (1168)

. . . . . Exon 28 . 7977A . . .

7901 ttcctcaggaacccaggggtccaggcccaagaccctcctcccgttttcttccagGAAGCCTCCATCTCAAAGGCAA*G*CTCATTTTTGGGGGAGAAAGCAA 8000

(1169) E A S I S K A **S** S F L G E K A S 1184

**N**1176 (*Ch6/Rg3*)

*Xcm*I RFLP (Rg1)

. . . . 8052T .8065G T8070 TC8080 . .

8001 GTGCTGGGCTCCTGGGTGCCCACGCAGCTGCCATCACGGCCTATGCCCTGA*C*ACTGACCAAGGC*C*CCTG***C****G*GACCTGC***GG***GGTGTTGCCCACAACAACCT 8100

A G L L G A H A A A I T A Y A L **T** L T K A P **A** D L **R**  G V A H N N L 1217

1191**S**  1205A **V**1207 **L**1210 (Ch1/Rg1 determinant)

Splice junction mutation D1226, cryptic splice site, fs4, **1230x**; E94,SS, *HLA A30 B18 DR7*

. . **a** . 8140c +c8144 . . -c8172 . -c8182 . .

8101 CATGGCAATGGCCCAGGAGACTGGAG***g***tgaggg*gt*gagg*g*gct*c*tggcagtgagcctgaggcccaggggac*c*ttaggatcc*c*tgagtgtgcccagaggga 8200

M A M A Q E T G **D** (1226)

. . . . . . . . . .

8201 gaggctggatgaagactcagaggaggaatgaagttataagcaggggtgggttgggggagactcaggagagcccagcagggggtggctaagggccagggga 8300

. . . . . Exon 29 8372**+TC** 2-bp insertion S1232 fs75, **1307x**

8301 ccaggctcttctccctgccttcctgtttactcgtggtctcccttcactttcagATAACCTGTACTGGGGC**TC**AGTCACTGGTTCTCAGAGCAATGCCGTG 8400

(1227) N L Y W G **S** V T G S Q S N A V 1241

1236 | C3b binding site

8412A . . . . . . . . .

8401 TCGCCCACCCC*G*GCTCCTCGCAACCCATCCGACCCCATGCCCCAGGCCCCAGCCCTGTGGATTGAAACCACAGCCTACGCCCTGCTGCACCTCCTGCTTC 8500

S P T **P** A P R N P S D P M P Q A P A L W I E T T A Y A L L H L L L H 1275

P1245

. . . T8533 . . . . . . .

8501 ACGAGGGCAAAGCAGAGATGGCAGACCAGGCT*G*CGGCCTGGCTCACCCGTCAGGGCAGCTTCCAAGGGGGATTCCGCAGTACCCAAgtaggggccgtccc 8600

E G K A E M A D Q A **A** A W L T R Q G S F Q G G F **R** S T Q (1303)

**S**1286 **V**1300 (protein sequencing)

.g8611 . . . . . Exon 30 1307 . . .

8601 cgggctctgg*c*gggggtgggtagtcctcagaccaagggcttgcttgagtcctggctcaacctccctagGACACGGTGATTGCCCTGGATGCCCTGTCTGC 8700

(1304) D **T** **V** I A L D A L S A 1314

1305**G** **G**1306 (protein sequencing)

8708T . . . . . . . . . .

8701 CTACTGG*A*TTGCCTCCCACACCACTGAGGAGAGGGGTCTCAATGTGACTCTCAGCTCCACAGGCCGGAATGGGTTCAAGTCCCACGCGCTGCAGCTGAAC 8800

Y W **I** A S H T T E E R G L N V T L S S T G R N G F K S H A L Q L N 1347

**F**1317 (glycosylation-BAC) N1328 **C4d ends** 🡨1336 | factor I cleavage site

**CUBf**: 1324-1388

. . . . . . . . . 8898a .

8801 AACCGCCAGATTCGCGGCCTGGAGGAGGAGCTGCAGgtgaaccactccctggtgaaccactccctcgcctgggtagccaggacacctgggcctcgtg*g*cc 8900

N R Q I R G L E E E L Q (1359)

. a8920 . . . . . . . .

8901 aggccagaagccgtcccca*c*cctcccacccgtggaatccccgcagcacttcttcctggggtcttcgggggaagactgacttcctggctgcgtgacctgga 9000

. . . . . . . . . .

9001 gctctgagcttcagttttctcacttgtagagtaacatacacagagttcaccctacagggtcgttagaaggctgaagtgagataattcatgtgctggtata 9100

. . .g9131 +c9139 . . c9164 . . . .

9101 aactttgtggaaatgtgaggtggggagagg*a*ggtgggg*c*tgttttgaggaaggagataagtta*t*tggagccgcaaaaacaggtttgcttgtgcccttcta 9200

. +c 9219 Exon 31 . . . . . . .

9201 acatcgccttcccttttc*t*gttgctgaagTTTTCCTTGGGCAGCAAGATCAATGTGAAGGTGGGAGGAAACAGCAAAGGAACCCTGAAGgtgagggccag 9300

(1360) F S L G S K I N V K V G G N S K G T L K (1379)

. . . . . . . . . .

9301 ggaaggggtggggccaggcactggtggaggagagggtgtggagtgagaggcctgtgggcagaggcacatggtccggggaaggaggcagacacctcagggt 9400

. . . . . . . . . .

9401 tggtgtcccgtgcttccgtcctgggtgtttttccccctgcttgctttcgcttgctctccccatctctgggtacctgttgtttcctttacccgcctcagtg 9500

. . . . . . . . . .

9501 ctggtggctccgaatcccactcctcagcccaggcctcttccctgaaccatgggccccactcgtcccactcccacagcacctcagacgaggcatgtcccaa 9600

. . . . . . . . . .

9601 agcccttcttcattctgtgtctcttgtctggctggtgggagcccctcccagccaggagcccagccactactctagaggccgtgttagtggcccctctccc 9700

. . . . . . . . . .

9701 aagcctgtccttatgtccctagtgactcctcctctgctcccctgctgcctgtggcccttggtgctgcatcctagattctgtgctgagacggccttctccc 9800

. . . . . . . . . .

9801 tacctggaacttctctctacctcctgtctcccctgtctgatccactgtccacacggcagtgacactgaccttccaaaagccccagccagatcagccttgg 9900

. . . . . . . . . .

9901 ggaaaagtcactccccgctgcccacggctcagatggctgggcctctgcccacccctccggccagacagctctccttgtctacacagatccccttgccttt 10000

. . . . . . . . . .

10001 cctgtccttccctgcttcttggcccacaggacaagctctttcttctccttcaagccttggccagaagcctttcctgagcttttcagtccagcctcttccc 10100

. . . . . . . . . .

10101 agcacagtctggagtgttggcctctgggggcaggcccctgcttctttacctctctgtctcgcctgacgcctgtggcgaatgtggtgccactcgtgtgtgt 10200

. . . . c10248 . . . . .

10201 ggactgtgcagtgacggggaggaaaaggggctgaaggcctcaaatcc*t*gtagcccagggagatgcccttaggtatggcaccagagaggtctgtggcctca 10300

. . . Exon 32 . . . . . .

10301 catgtcccacgtcctctccctgccccttgctgagccagGTCCTTCGTACCTACAATGTCCTGGACATGAAGAACACGACCTGCCAGGACCTACAGATAGA 10400

(1380) V L R T Y N V L D M K N T T C Q D L Q I E 1400

1389-1427: **MG8α** N1391 (glycosylation-BAC)

. . . . . . . . . .

10401 AGTGACAGTCAAAGGCCACGTCGAGTACACGAgtgagtgtgggggttgggaggccttggggccaggcaggggctggcgcagggagccgggtggccatccc 10500

V T V K G H V E Y T M (1411)

. . Exon 33 . 10550---------10559, deleted in C4A4? . .

10501 agccctcctcacaatgcttccctgtgcagTGGAAGCAAACGAGGACTAT*GAGGACTAT*GAGTACGATGAGCTTCCAGCCAAGGATGACCCAGATGCCCCT 10600

(1412) E A N **E D Y (E D Y) E Y D E** L P **A** K D D P D A P 1434*

1417-sulfation sites 🡨|1427 α chain ends (plasma form)

1420 1422

. . . . . . . . . .

10601 CTGCAGCCCGTGACACCCCTGCAGCTGTTTGAGGGTCGGAGGAACCGCCGCAGGAGGGAGGCGCCCAAGGTGGTGGAGGAGCAGGAGTCCAGGGTGCACT 10700

L Q P V T P L Q L F E G **R R** N **R R R R** E A P K V V E E Q E S R V H Y 1468

1449| 1454 |🡪 γ chain

**MG8γ**:1454-1573

. . . . c? . . . . . .

10701 ACACCGTGTGCATCTGgtgggcgccgggagctgccctgggccaggg*g*agggagggcaggacccaggctggggctgggcttctggagcccgcgcaggcaga 10800

T V C I W (1473)

. . .Exon 34 . . . . . . .

10801 acctggacgacagctcacacgtctccacagGCGGAACGGCAAGGTGGGGCTGTCTGGCATGGCCATCGCGGACGTCACCCTCCTGAGTGGATTCCACGCC 10900

(1474) R N G K V G L S G M A I A D V T L L S G F H A 1496

10910T . . . . . . . . .

10901 CTGCGTGCT*G*ACCTGGAGAAGGTGTGgtcagccacccagggcaaccccctctgtcccaggtactgagccctgtcatgtgcagggcctgtgaccaactccc 11000

L R A **D** L E K (1503)

**Y**1500

. Exon 35 . . . . . . . . .

11001 cttttccacagCTGACCTCCCTCTCTGACCGTTACGTGAGTCACTTTGAGACCGAGGGGCCCCACGTCCTGCTGTATTTTGACTCGgtgagtggggagag 11100

(1504) L T S L S D R Y V S H F E T E G P H V L L Y F D S (1528)

. . . . . . . . . .

11101 atgaggcaggaagggactcgatggcaccgggtttactgagtatgcgttaggaggtttctcaggagacagctgtgtcagcggctggtgctcttgagaactt 11200

. . . . . . . . . .

11201 gtgatgtcatcagagagaaggacaagaatgtgagcccgtgagacacagcagagtaaggggcagacctgcaggcggcagggaccgatgccagtcagcaggg 11300

. . . . . . . . . .

11301 accctcagggtttgagagggagtctttcctaatgctggttttattcagcttgaggggctgcctttgtttttttgttgaacttcctatcttttttttaata 11400

. . . . . . . . . .

11401 ttaaagcgtattttcctttacaaagtgatggtggccatagatgatagttgtatttgtcttttcacgaccttatttggctaaaatagttatcaaccctctt 11500

. . . . . . . . . .

11501 acggctctcaaaacatttttatttatttatttagtaaagacagggtctcgctctgttgcccaggctggtcttgaactcccggcctcaagcgatcctctgg 11600

. . . . . . . . . .

11601 cctaggcctttcaaagtaccggatttacaggccagagccaccatgcccggccttcaaaaaaagttttggaacatttactgtaacctctgggagaaaatgt 11700

. . . . . . . . . .

11701 gagaaaggtgtggtggctgtcattagccagctgtttgtaggtcagggagacccctacccagtgtgtgcagaggggccagcccccatcagctggggaagcc 11800

. . . . . . . . . .

11801 tggctgacacatctgggttgaacacaatagaaaacacagagccaacaagattcccggatagggagctgacggtgcagcagcctagctcaggagggacact 11900

. . . . . . . . . .

11901 ggcacggcaccgtgtggactgggcccgcgtgggcacgaggaggggtcaggcctgggacctgagtcggggggtcaggcaggatgacagaacctgcagttag 12000

. . g12028 . . . . . . .

12001 gttgtggcaaataaaggaggacccagt*t*gtatccatgacaaagatgaggccgcgaggagggcgagtgggtttgggggcaggcagagtgccttggagaact 12100

. . . . . . . . . .

12101 tacaggtcctgccacaatcctaatgcaaggatggagctgcaagttcagtttgggaatcatcagcctggattggtttggtggaagccagggagtggttgag 12200

. . . . . . . . . .

12201 acccccacaggggagctctgaggaaggaagttccgaaggagggaacgtaagaaatgaccaggtcagaaccaagggtggtccagaagctaacccttagctt 12300

. . . . . . . . . .

12301 agggacagtttcacagagaacacgtccatgatgcaagactctgctgagggcctggagcagtgaagactggggcaaggtcaccctctgggaagtgaagtca 12400

. . . . . . . . . .

12401 ccagagaccttgcggagcagctttgagagttctctgagtaggaaggtaacagaatgtgaaggacactggagagaaggccaataggaagcaaacaaaaaca 12500

. . . . . . . Exon 36 . .

12501 ggccaaggaaacccagtacagggggctgcagggcccagggagtgggtccctcatctctcctccccacgcttggccagGTCCCCACCTCCCGGGAGTGCGT 12600

(1529) V P T S R E C V 1536

. . . . . . **+GACT** 4-bp insertion, *HLA A1 B17 DR13*

12601 GGGCTTTGAGGCTGTGCAGGAAGTGCCGGTGGGGCTGGTGCAGCCGGCCAGCGCAACCCTGTAC**GACT**ACTACAACCCCGgtgagcactgcaggacaccc 12700

G F E A V Q E V P V G L V Q P A S A T L Y D **Y** Y N P E (1563)

**Y1559x** (both C4A and C4B, LS)

. . . . . . . . . .

12701 tgaaattcaggagaactttggcataggtgccctcctatgggacaatggacaccggggtagtgagggggcagagagccctggggctccctgggactgagga 12800

. . . . Exon 37 . . . . .

12801 ggcagaatggaggggcctgtgccctaactcctctctgttctccagAGCGCAGATGTTCTGTGTTTTACGGGGCACCAAGTAAGAGCAGACTCTTGGCCAC 12900

(1564) R R C S V F Y G A P S K S R L L A T 1581

**Anchor**:1574-1594

. . . . . . . . . .

12901 CTTGTGTTCTGCTGAAGTCTGCCAGTGTGCTGAGGgtgagactgagggcctggggcggggcagtggaggcgggatggccggggccccccccacactgtct 13000

L C S A E V C Q C A E G (1593)

. .Exon 38 . . . . . . . .

13001 gatgggttccccaacttcagGGAAGTGCCCTCGCCAGCGTCGCGCCCTGGAGCGGGGTCTGCAGGACGAGGATGGCTACAGGATGAAGTTTGCCTGCTAC 13100

(1594) K C P R Q R R A L E R G L Q D E D G Y R M K F A C Y 1621

**C345C**:1595-1744

. . . . . . c13169 . . .

13101 TACCCCCGTGTGGAGTACGgtcagtcttcccaccgaggccctggcctgaccctccctcggggaccggc*t*gttttggtctctctgggtgtagcctgctcct 13200

Y P R V E Y G (1626)

. . . . . . . . . .

13201 cttacaggtcatgcacgcagcctgtttgctctgacaccaacttcctaccctctcagcctcaaagtaactcacctttcccccttctcctcaccccctctta 13300

Exon 39 . . . . . . . . . .

13301 gGCTTCCAGGTTAAGGTTCTCCGAGAAGACAGCAGAGCTGCTTTCCGCCTCTTTGAGACCAAGATCACCCAAGTCCTGCACTTCAgtatgaagcaaaccg 13400

(1627) F Q V K V L R E D S R A A F R L F E T K I T Q V L H F T (1654)

. . . . . . . . . .

13401 gagaggcgggcagggctggggggagacagggaggctgaggtgtggccgaggacctgaccatctggaagtgtgaaaatccccttgggctgtcagaagcctt 13500

. . . . . . . . . .

13501 gggcttggccataaatagggaggcagtggcacctctccatgggggtggcgaaggtggaatgagaggatctacacagagtccccagcctgggctcaccctg 13600

. . . . Exon 40 . . . . .

13601 caccttctcttcccctctgaccacttttgcgcacgtcatccccgcagCCAAGGATGTCAAGGCCGCTGCTAATCAGATGCGCAACTTCCTGGTTCGAGCC 13700

(1655) K D V K A A A N Q M R N F L V R A 1649

. . . . . . . . . .

13701 TCCTGCCGCCTTCGCTTGGAACCTGGGAAAGAATATTTGATCATGGGTCTGGATGGGGCCACCTATGACCTCGAGGGACAgtgagtcatctggtcccctc 13800

S C R L R L E P G K E Y L I M G L D G A T Y D L E G H (1698)

. . . . . . . . . .

13801 agtctcttgtcctccccatgcctcgccacctaggccttgcccctcagaagccagatgcctgtgctctccgtttccacctgccatcctcccgagccctgct 13900

. . Exon 41 . . . . . . .

13901 gactgcccctttgccccctgcagCCCCCAGTACCTGCTGGACTCGAATAGCTGGATCGAGGAGATGCCCTCTGAACGCCTGTGCCGGAGCACCCGCCAGC 14000

(1699) P Q Y L L D S N S W I E E M P S E R L C R S T R Q R 1724

. . . . . . . . . .

14001 GGGCAGCCTGTGCCCAGCTCAACGACTTCCTCCAGGAGTATGGCACTCAGGGGTGCCAGGTG**TGA**GGGCTGCCCTCCCACCTCCGCTGGGAGGAACCTGA 14100

A A C A Q L N D F L Q E Y G T Q G C Q V * (1744)

. . . . . . poly(A) signal. * poly(A) site (C4B)

14101 ACCTGGGAACCATGAAGCTGGAAGCACTGCTGTGTCCGCTTTCATGAACACAGCCTGGGACCAGGGCAT**ATTAAA**GGCTTTTGGCAGC**A**AAGTGTCAGTG 14200

* poly(A) site (C4A) . . . . . . . .

14201 TTGGC**A**gcgaagtgtcagtgtgtgttgctagggctgagagcagtgcccctgcccgatgcagttctgggcaggccaggttgacataaccttagactctctg 14300

**Figure S1.** An annotated human short *C4B* gene sequences at locus I of the *RCCX* module with structural domains and sequence variations or mutations*.* Information for C4 exon-intron structure was based on our earlier published sequences with accession numbers M59815, U07856 and M59816 for a *C4A* gene and with endogenous retrovirus HERV-K(C4) in intron 9 of long genes (reference 38). Intron 9 sequences for short *C4* genes were originally published in reference 37. Accession numbers and sequence information for *RP1/RP2* (or *STK19/STK19P*) upstream of C4 are L26260 to L26263, which were originally published in reference 35. The contiguous framework sequence was taken from GenBank sequence with accession number AL049547.10, starting from nucleotide 22941 of the contig. Besides providing the exon-intron structures with DNA and protein sequences plus major protein structural domains, this figure highlights genomic regions with polymorphisms and mutations for the two clusters of variations and mutations. One of them is present in a region spanning between exon 15 and exon 21 of a *C4* gene. This region encodes the junction for the β−α chains of C4A or C4B proteins, the anaphylatoxin-like C4a domain that is cleaved during activation of the classical and the lectin pathways, plus a biantennary complex protein glycosylation site. Notice that polymorphic sites responsible for the fastest (C4B7) and slowest (C4B96) migration of C4B are both present in this cluster of variations. The other cluster of sequence variations is present at the C4d region encoded by exon 23 to exon 30. This C4d region contains the thioester domain (TED), the C4A and C4B isotypic sequences and the Chido (Ch) and Rodgers (Rg) blood group antigenic determinants. Exon sequences are in upper cases. Intron sequences are in lower cases. Single letter codes are used for amino acid sequences. Variant nucleotide sequences are shown above reference DNA sequences. Polymorphic amino acid sequences are shown below reference protein sequences. Defining sites for restriction fragment length polymorphisms (RFLPs) are marked. This supplementary figure presents an updated and extended version of annotated human complement *C4* sequences from our earlier work shown in references 5, 35, 37 and 38.

(Figure S2 on next page)

**Fig. S2**. **Activations and regulations of complement pathways**. The complement system is designed to be a key defensive armament against infections for both innate and adaptive immune responses. It is a set of highly sophisticated tools that lyses microbial cells through the formation of membrane attack complexes (MACs) that detrimentally pierce through target membrane to form multiple pores. However, they exert minimum damage to the self or autologous host cells under normal conditions. Activation products of the complement system summon inflammatory cells to migrate to the site of microbial invasion to assist performing the defense task, a process known as anaphylaxis. Covalent depositions of activated complement proteins on microbes help solubilizing immune aggregates, facilitate the clearance immune complexes (IC) in the circulation by red blood cells by complement receptor CR1, and opsonize IC for phagocytosis through binding to complement receptors expressed on myeloid cell membranes. Complement activation starts by recognition of foreign objects through three different pathways or strategies to generate an initiation complex. The initiation complex produces active subunits for C3 convertase, which are C4b2a (i.e., C4bC2a) for the classical pathway (CP) and the lectin pathway (LP), and C3bBb for the alternative pathway (AP). The C3 convertase cleaves C3 to C3a and C3b. An association of C3b with either of the convertases changes the enzyme specificity from C3 to C5. The C5 convertase cleaves C5 to C5a and C5b. Both C3 convertase and C5 convertase are serine proteinases. The enzymatic subunits are present in C2a for the lectin and classical pathways, and Bb for the alternative pathway. The smaller moieties of activation products for the convertases, C3a and C5a, are potent anaphylatoxins that attract inflammatory cells to the sites of complement activation and stimulate inflammatory cell to release secretary granules. Anaphylatoxins also increase the permeability of endothelial linings for blood and lymphatic vessels to facilitate infiltrations of inflammatory cells into tissues. The larger moieties, C3b and C5b, stay close to the target surface. C5b is a seeding molecule for the bindings of C6 and C7. The resultant trimolecular structure, C5b-C6-C7 (i.e., C5b-7), penetrates into the target membrane, attracts the association of C8 and promotes the polymerization of C9 to form a tunnel-like structure (C5b-9_n_) across the target membrane to destroy the invading microbe. The progression of activation and terminal pathways are shown in red arrows. Regulations by dissociations and blockage are shown by unfilled green arrows; proteolytic degradations of C4b and C3b are shown by solid green arrows. A positive feedback mechanism to generate C3b is shown by blue dotted lines. The location of inhibition by eculizumab in the terminal pathway is shown by purple diamond arrows. (Taken from reference 2)
