# Supplementary material for: Human Complement C4B Allotypes and Deficiencies in Selected Cases With Autoimmune Diseases
Source: Front Immunol. 2021 Oct 26;12:739430. doi: 10.3389/fimmu.2021.739430 (PMC8577214; doi:10.3389/fimmu.2021.739430)
Supplement: Supplementary file 2 [file Table_1.docx]

**Table S1. A summary of landmark structures and polymorphic variants in human complement *C4* genes (June 30, 2021)**

| *C4B* gene ref DNA position | Ref DNA sequence | Variant Sequence | Position | Amino acid sequences | remarks |
| --- | --- | --- | --- | --- | --- |
| - 51 |  |  | exon 1 |  | transcriptional initiation |
| 1 to 57 |  |  | exon 1 |  | M1 to Q19, leader peptide |
| 58-60 |  |  | exon 1 | K20 | N-terminus, mature protein; β-chain |
| 116 | g | a | intron 1 |  | tcgg/tcga *Taq*I RFLP |
| 378 | C | T | exon 2 | F82F | synonymous |
| 2296 | C | A | exon 9 | S347Y | S328Y |
| 2371 | g | a | intron 9 |  |  |
| 2558 | g | c | intron 9 |  |  |
| 2564 | g | a | intron 9 |  |  |
| 2572-77 |  |  | intron 9 |  | cagaca-integration site, human endogenous retrovirus HERV-K(C4); long gene add 6367 bp at nucleotide 2578 |
| 2577 | t | c | intron 9 |  |  |
| 2592 | g | a | intron 9 |  |  |
| 2601 | c | t | intron 9 |  |  |
| 2651 | t | c | intron 9 |  |  |
| 3024 | T | C | exon 11 | V418A |  |
| 3345 | C | T | exon 12 | R477W | C5 binding site; C4A6 |
| 3349 | C | T | exon 12 | P478L | C5 binding site; hemolytically inactive C4B1 |
| 3463 | c | a | intron 12 |  |  |
| 3594 | GT | Δ | exon 13 | V516 fs110, 626x | ΔGT 3594; Austrian/Italian  *HLA A24 B38 Cw7 DR13* |
| 3671 | C | Δ | exon 13 | F541 ΔC, fs47, 588x | C4BQ0 in LS, stops at codon 588; C4A had 2-bp insertion at codon 1232; French  *HLA A2 B12 DR6* |
| 3723 (10147) | C | T | exon 13 | R559x | CGA🡪TGA; C4A, mono-long; Moroccan family  *HLA A2 B17 DR7* |
| 4312 | − | tt | intron 14 |  | tt-insertion |
|  |  |  | exon 15 | C635S | C635S; protein sequencing |
| 4432 | G | A | exon 15 | W660x | W660x; E133; stops at codon 660; recurrent; East Asians  *HLA B*15:27, C*04:01, DRB1*04:06* |
| 4645-56 |  |  | exon 16 | RKKR 677-680 | β−α chain junction |
| 4657 |  |  | exon 16 | N681 | amino terminus, α-chain; C4a starts |
| 5056 | C | T | exon 17 | P726L | *Pvu*II RFLP; cos 3A3 |
| 5065 | G | A | exon 17 | R729Q | C4B7; HC74; *Nci*I RFLP |
| 5142 | C | Δ | exon 17 | Q755, fs12, 767x | 5142 ΔC, stops at codon 767; MS630 |
| 5793 | T | C | exon 20 | V825V | V806V |
| 5809 | C | Δ | exon 20 | R831, fs32, 863x | R831 ΔC; stops at 863; Swedish  *HLA A30 B18 DR3* |
| 6077 | G | T | exon 21 | G882G | synonymous |
| 6150 | A | G | exon 21 | T907A |  |
| 6189 | G | A | exon 21 | E920K | C4B96; *Eco*RI RFLP |
| 6272 | c | t | intron21 |  |  |
| 6331 | a | g | intron 21 |  |  |
| 6419 | t | c | intron 21 |  |  |
| 6642-7 |  |  | exon 23 | R-T, 956-957 | factor I cleavage site, C4d starts |
| 6775 | c | t | intron 23 |  |  |
| 6939-50 |  |  | exon 24 | CGEQ 1010-1013 | thioester bond |
| 7022 | G | T | exon 24 | L1037L | synonymous |
| 7292 | G | T | exon 25 | A1068V |  |
| 7308 | G | A | Exon 25 | G1073D | formerly D1054G; Ch5; D1054 in C4B2, B5 and B7 |
| 7318 | C | T | exon 25 | T1076T | synonymous |
| 7335 | C | A | exon 26 | G1095G | synonymous |
|  |  |  | exon 26 | S1109I | Protein sequencing |
| 7609 | T | C | exon 26 | L1120P | C4B/C4A isotypes |
| 7613 | C | G | exon 26 | S1121C | C4B/C4A isotypes, *Psh*AI RFLP |
| 7620 | A | T | exon 26 | I1124L | C4B/C4A isotypes |
| 7623 | C | G | exon 26 | H1125D | C4B/C4A isotypes |
| 7625 | T | C | exon 26 | H1125D | C4B/C4A isotypes |
| 7977 | G | A | exon 28 | S1176N | Ch6/Rg3 determinant |
| 8052 | T | C | exon 28 | T1191S |  |
| 8065 | C | T | exon 28 | A1205A |  |
| 8070 | C | G | exon 28 | A1207V | ADLR 1207-1210 VDLL; Ch1/Rg1; *Xcm*I RFLP  C4A1, reverse association with ADLR |
| 8080 | GG | TC | exon 28 | R1212L | ADLR 1207-1210 VDLL; Ch1/Rg1;  C4B5, reverse association with VDLL |
| 8127 | g | a | intron 28 | D1226, fs4, 1230x | gt-->at, splice junction mutation, cryptic splice site; stops at 1230; C4B-C4B; SS; E94, C4DF1, C4DF3; Europeans  *HLA A30 B18 DR7* |
| 8140 | g | c | exon 28 |  |  |
| 8144 | c | cc | intron 28 |  | c-insertion |
| 8172 | c | Δ | intron 28 |  | c-deletion |
| 8182 | c | Δ | intron 28 |  | c-deletion |
| 8332 | c | t | intron 28 |  |  |
| 8371-72 | TC | TCTC | exon 29 | S1232, fs75, 1307x | 2-bp insertion; recurrent; C4A mutations; stops at 1307 |
| 8412 | G | T | exon 29 | P1245P | synonymous |
| 8533 | G | T | exon 29 | A1286S |  |
| 8575-7 |  |  | exon 29 | R1300V | protein sequencing |
| 8611 | c | g | intron 29 |  |  |
| 8676 |  |  | exon 30 | V1306G | protein sequencing |
| 8708 | A | T | exon 30 | I1317F |  |
| 8767 |  |  | exon 30 | R-N 1336-7 | C4d ends; factor I cleavage site |
| 8898 | g | a | intron 30 |  |  |
| 8920 | c | a | intron 30 |  |  |
| 9131 | a | g | intron 30 |  |  |
| 9139 | c | cc | intron 30 | insertion |  |
| 9164 | t | c | intron30 |  |  |
| 9219 | t | c | intron 30 |  |  |
| 10248 | t | c | intron 31 |  |  |
| 10541-70 |  |  | exon 33 | EDYEDYEYD 1415-24 | tyrosine sulfations |
| 10595-7 |  |  | exon 33 | A1433 | C-terminus, plasma form α-chain |
| 10645-57 |  |  | exon 33 | RRRR 1450-3 | α−γ chain junction; γ-chain starts at 1454 |
| 10747 | g | g/c | intron 33 |  |  |
| 10910 | G | T | exon 34 | D1500Y | single protein variant in γ-chain |
| 12028 | t | g | intron 35 |  |  |
| 12668  (19035) | GACT  GACT | GACTGACT  GACTGACT | exon 36  exon 36 | Y1559x  Y1559x | 4-bp, GACT insertion; Y1559 to stop; LS: C4A-C4B, identical mutations; Algerian family  *HLA A1 B17 DR13* |
| 13169 | t | c | intron 38 |  |  |
| 14063-5 |  |  | exon 41 | V1744 | 1745 TGA-stop codon; V1744 carboxyl-terminus, γ-chain / C4 protein |
| 14170-5 |  |  |  |  | ATTAAA,-poly(A) signal |
| 14189 |  |  |  |  | poly(A) site - C4B |
| 14206 |  |  |  |  | poly(A) site - C4A |
|  |  |  |  |  |  |

^*^ Single letter representations for amino acids are shown.

^¶^ The first nucleotide of the initiation codon for the *C4* gene is assigned as number 1 for *C4* genomic sequences.
